# Diagnostic and Predictive Applications of Functional Near-Infrared Spectroscopy for Major Depressive Disorder: A Systematic Review

**DOI:** 10.3389/fpsyt.2020.00378

**Published:** 2020-05-06

**Authors:** Cyrus S. H. Ho, Lucas J. H. Lim, A. Q. Lim, Nicole H. C. Chan, R. S. Tan, S. H. Lee, Roger C. M. Ho

**Affiliations:** ^1^Department of Psychological Medicine, Yong Loo Lin School of Medicine, National University of Singapore, Singapore, Singapore; ^2^Department of Psychology, Faculty of Arts and Social Sciences, National University of Singapore, Singapore, Singapore; ^3^Institute of Health Innovation and Technology (iHealthtech), National University of Singapore, Singapore, Singapore

**Keywords:** diagnostic, prediction, functional near-infrared spectroscopy, major depressive disorder, systematic review

## Abstract

**Introduction:**

Major depressive disorder (MDD) is a global psychiatric disorder with no established biomarker. There is growing evidence that functional near-infrared spectroscopy (fNIRS) has the ability to aid in the diagnosis and prediction of the treatment response of MDD. The aim of this review was to systematically review, and gather the evidence from existing studies that used fNIRS signals in the diagnosis of MDD, correlations with depression symptomatology, and the monitoring of treatment response.

**Methods:**

PubMed, EMBASE, ScienceDirect, and Cochrane Library databases were searched for published English articles from 1980 to June 2019 that focused on the application of fNIRS for (i) differentiating depressed versus nondepressed individuals, (ii) correlating with depression symptomatology, and in turn (iii) monitoring treatment responses in depression. Studies were included if they utilized fNIRS to evaluate cerebral hemodynamic variations in patients with MDD of any age group. The quality of the evidence was assessed using the Newcastle–Ottawa quality assessment scale.

**Results:**

A total of 64 studies were included in this review, with 12 studies being longitudinal, while the rest were cross-sectional. More than two-thirds of the studies (n = 49) had acceptable quality. fNIRS consistently demonstrated attenuated cerebral hemodynamic changes in depressed compared to healthy individuals. fNIRS signals have also shown promise in correlating with individual symptoms of depression and monitoring various treatment responses.

**Conclusions:**

This review provides comprehensive updated evidence of the diagnostic and predictive applications of fNIRS in patients with MDD. Future studies involving larger sample sizes, standardized methodology, examination of more brain regions in an integrative approach, and longitudinal follow-ups are needed.

## Introduction

Major depressive disorder (MDD) is a global mental illness which is increasingly prevalent in modern societies. As per the World Health Organization, MDD affected approximately 322 million people of all ages globally. The total number of people likely to have depression increased by 18.4% between 2005 and 2015, and this number is expected to increase exponentially over time (1). Symptoms of depression include low mood, decreased energy, poor attention, memory problems, disturbed appetite and sleep, anhedonia, feelings of guilt, and worthlessness (2). In severe cases, depression may present with psychotic symptoms, suicidal thoughts and increase the possibility of unnatural death (3). MDD can also cause significant disability. Depression is one of the leading cause of world disability in the year 2020, and in 10 years' time, it is anticipated to be the biggest cause of global disease burden overtaking cardiovascular diseases (1). Despite its gaining prevalence, MDD is still considerably undertreated and under diagnosed, especially in primary care settings (4).

Depressive disorders have been correlated with problems in the limbic, thalamic and cortical areas (5). MDD has also been correlated with neuropsychological deficiencies in numerous cognitive areas, involving attention, language, memory, and executive function (6). To diagnose patients with MDD, clinicians conventionally refer to the International Statistical Classification of Diseases and Related Health Problems—10^th^ revision (ICD-10) or the Diagnostic and Statistical Manual of Mental Disorders, 5th Edition (DSM-5) classifications as guides to confirm the diagnosis. However, the accuracy of reaching a diagnosis of MDD relying on history taking remains debated. Diagnosis is often based on the subjective assessment and clinical experience of the clinicians. Some patients may also not be forthcoming about their symptoms, especially suicidal ideation. Furthermore, many of the psychiatric symptoms are polymorphous and may overlap in various psychiatric disorders, thereby making diagnosis all the more challenging.

MDD is a multifaceted and varied illness in which up to two thirds of patients could experience treatment resistance that protracts and worsens the episodes (7). Only approximately 33% of patients with MDD attain remission, despite being treated with optimal medications based on measurement-based care and consensus guideline. Furthermore, the probability of treatment response seems to decrease with each new treatment option (8). Treatment-resistant depression (TRD) is related to increased morbidity and mortality, with recurring and chronic periods in the long run (9). Hence, it would be useful if there are ways to ascertain improvements in treatment at any stage of the illness, as it would offer wider benefits for global management of depression.

Biomarkers offer a conceivable target for assisting in the diagnosis and identifying predictors of response to various interventions (10). Biomarkers may come in various forms, such as inflammatory markers, endogenously produced hormones and brain imaging. In recent years, brain-imaging techniques such as electroencephalography (EEG), functional magnetic resonance imaging (fMRI), positron emission tomography (PET), and magnetoencephalography (MEG) existed to be used as adjuncts to help clinicians diagnose MDD. According to some neuroimaging findings using PET and fMRI to investigate brain function in patients with depression (11–13), they found blood flow reduction in the prefrontal cortex to be associated with a decline in activity within the cingulate cortex. There had been reports using neuroimaging techniques recording hemodynamic response relating to brain activity during cognitive stimulation on depressed patients. One such study using fMRI performed by Okada et al. revealed decreased left prefrontal activation and reduced task performance in depressed patients utilizing the verbal fluency task (VFT) (11). However, these tests are expensive to conduct. Additionally, the patients were required to place themselves in an awkward posture, i.e., lying supine in a narrow space with the head fixed during the investigation (14).

In 2009, functional near-infrared spectroscopy (fNIRS) was sanctioned in Japan. It was classified to be an advanced medical technology for differentiation of psychiatric illness (15). Additionally, in 2013, fNIRS obtained medical insurance coverage for being an adjunct diagnostic tool. fNIRS examinations have since been utilized for psychophysiological assessment of cognitive function.

fNIRS is a form of spectroscopy that utilizes light sources between a spectral window of 650 to 1000 nm which penetrates organic tissues. Oxygenated hemoglobin (oxy-Hb) variations are then computed using the variance in absorbance using the modified Beer–Lambert law. This is a noninvasive technique that can detect cortical oxygenation levels of hemoglobin (16), and low cortical oxygenation levels have been correlated with depressive illness. The advantages of using fNIRS are that it is a relatively inexpensive procedure, portable, and easy to set up, and it does not involve nonionizing radiation. Hence, it may be repeated multiple times on an as-needed and when-needed basis for patients. In fact, fNIRS has been applied in many other areas of the medical field, such as cognition and preoperative functional assessment (17). With regard to psychiatric illness, in addition to MDD, there are studies pertaining to its use for patients with schizophrenia and bipolar disorder among other disorders (18) (**Table 1**). Many fNIRS studies involve tasks that help activate brain activity in the subject, such as VFT or passively viewing photographs to trigger an emotional response.

**Table 1:** Studies using fNIRS to assess different psychiatric disorders.

Study	Psychiatric disorder	Key finding
Noda T. et al. (19)	Schizophrenia	Prefrontal and temporal region oxy-Hb uptake during the post-verbal fluency task (VFT) period was associated with working memory deficits in patients with schizophrenia.
Hirose T. et al. (20)	Bipolar disorder	Suicide risk in patients with bipolar disorder was correlated with delayed activation timing of NIRS signal during the VFT in the prefrontal region.
Katzorke A. et al. (21)	Dementia/cognitive impairment	Patients with mild cognitive impairment had decreased hemodynamic response in the inferior frontotemporal cortex as compared to healthy controls using VFT.
Ueda S. et al. (22)	Attention-deficit hyperactivity disorder (ADHD)	Adult ADHD patients had reduced prefrontal hemodynamic response during the Stroop Color–Word Task compared to healthy controls, and this response was similar to pediatric studies.

Oxy-Hb, oxygenated hemoglobin; fNIRS, functional near-infrared spectroscopy.

To date, most of the studies on the diagnostic and predictive applications of fNIRS for MDD have been conducted in Japan. Furthermore, most studies have been conducted with a relatively small number of participants. To date, there is only one meta-analysis, published in 2015, looking at fNIRS for differentiating patients with depression from healthy subjects (23). Since then, more studies have been conducted on the matter as the use of fNIRS on depressed individuals to help in diagnosis and monitoring treatment response has gained acceptance over the last few years. Through a meta-analysis, Zhang et al. found that MDD patients had considerably decreased prefrontal cortex activation when undertaking cognitive tasks relative to controls. Patients with MDD, as opposed to controls, were associated with reduced rise in oxy-Hb in prefrontal regions during cognitive stimulation. This distinctive pattern of blood oxygen variations in the prefrontal cortex in MDD patients may be used as an objective diagnostic instrument for MDD. However, with such a low quantity of studies in the first meta-analysis, we decided to evaluate the use of fNIRS as a diagnostic biomarker for the diagnosis of MDD and its distinction between different stages of the illness, and to discuss its usefulness as a monitoring biomarker for treatment response in MDD patients, as based on a systematic review of the latest available literature. This paper aims to cover (i) the use of fNIRS to differentiate depressed from healthy individuals, (ii) correlation of fNIRS signals with depression symptomatology, and (iii) how it can be applied to monitor treatment response.

## Methods

### Data Sources and Search Strategy

This study was conducted on the basis of the Preferred Reporting Items for Systematic Reviews and Meta-Analysis (PRISMA). A systematic review was completed with published English-language literature from 1980 to June 2019, focusing on the utility of fNIRS for (i) differentiating depressed versus nondepressed individuals, (ii) correlating with depression symptomatology, and (iii) monitoring treatment responses in depression. The four electronic databases searched were PubMed, EMBASE, ScienceDirect, and Cochrane Library. The search terms used were “Spectroscopy” or “Near-infrared” or “near-infrared spectroscopy” or “fNIRS” or “optical topography” and “Depression” or “depressed” or “depressive disorder” or “mood disorder” or “affective disorder.” Terms were searched as both text words and subject headings. The team scanned for related publications, conference proceedings and bibliographies of papers gathered manually. This systematic review was funded by the National University of Singapore iHeathtech Other Operating Expenses (R-722-000-004-731).

### Eligibility Criteria and Data Collection

Studies were short-listed if the authors utilized fNIRS to measure cerebral hemodynamic variations in patients with MDD of any age group. All titles and abstracts retrieved from the databases were independently reviewed by two reviewers (LL and NC). Where appropriate, full-text papers were extracted for further inspection. Studies which were selected by either reviewer and fulfilled the inclusion criteria proceeded on to full-text review, whereby the study characteristics and results were extracted. Any discrepancies in the study selection were brought to the attention of the third reviewer (CH) and resolved by discussion. Studies were divided into the three categories that were most appropriate for the three questions set out in the review. The data review form consisted of the subsequent data: authors and publication year, country, sample characteristics, diagnostic criteria used, type of NIRS device, paradigm used, and main findings of the study. As much data as possible were obtained from the articles, and efforts were made to contact the authors if supplementary data were needed.

### Quality Assessment of the Articles

The articles included in the full-text review were evaluated by utilizing the Newcastle–Ottawa quality assessment scale (NOS) (24), with the subsequent generation of a table with star scores for each study. There is no overall score that determines whether a study is “good” or “bad,” but a star is awarded for meeting each criterion involving selection and outcome, with the exception of compatibility where two stars can be awarded. Two reviewers (LL and NC) independently assessed the papers, and discrepancies were brought to the third reviewer (CH) to resolve by discussion.

## Results

### Study Selection

**Figure 1:**
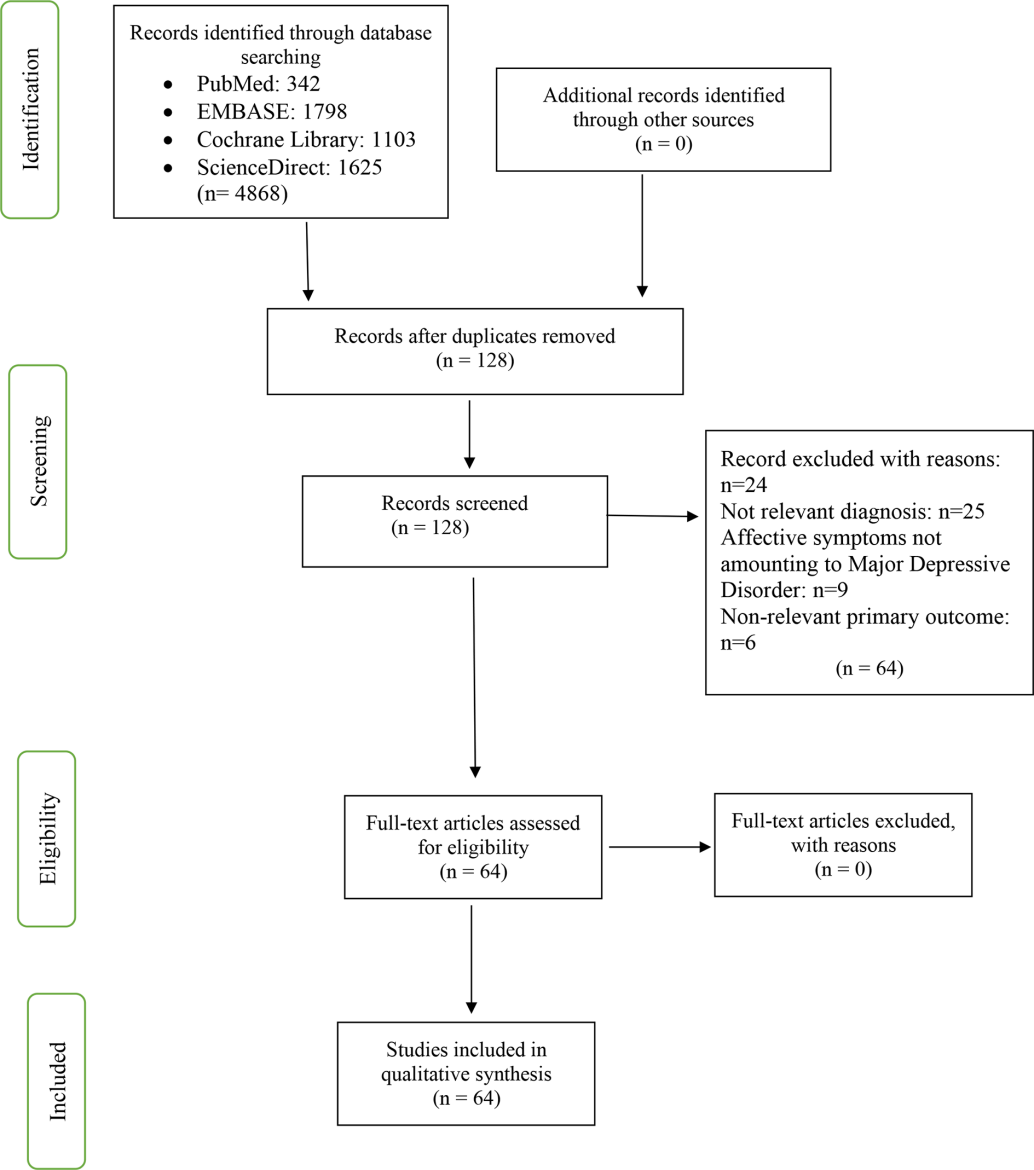
Flow diagram illustrating the literature search.

A total of 4868 citations were identified from our database search, with 342 from PubMed, 1798 from EMBASE, 1625 from ScienceDirect, and 1103 from Cochrane Library. After reviewing the titles, abstracts, and removing duplicated publications, 128 articles were selected. Of these, 64 studies met the inclusion criteria and were encompassed in this analysis. The selection process is displayed in **Figure 1**, constructed according to the PRISMA statement. Forty-seven studies were from Japan, eight studies from China, six studies from Germany, one study from the UK, one study from the USA, and one study from Uzbekistan. These studies were further divided into categories based on which of the three questions they addressed: (i) differentiation of depressed from healthy individuals, (ii) correlation with depression symptomatology, and (iii) assessment of treatment response. Fifty-one studies addressed the first question, 31 studies addressed the second question, and 16 studies addressed the third question. Twelve studies were longitudinal, while the rest were cross-sectional studies. Seven studies were extracted from conference proceedings, while the remaining were full-text articles.

### Use of fNIRS to Differentiate Depressed From Healthy Individuals

We identified 51 papers that used fNIRS to distinguish depressed patients from healthy controls (HCs) (**Table 2**). The bulk of the studies were conducted in Japan (n = 38), followed by China (n = 7), Germany (n = 4), and the United Kingdom (n = 1). The pooled sample across the studies comprised a total of 2094 unipolar depressed patients and 2457 HCs. All the depressed patients in the respective studies fulfilled the ICD-10 or DSM-IV diagnostic criteria for MDD, with the exception of two studies that used the DSM-5 and one study that used the DSM-III as the diagnostic criteria instrument. The psychopathology measure used for the studies was mostly the Hamilton Depression Rating Scale (HAMD). With the exception of six studies that did not include an indication of the medications that were consumed by the depressed patients, three studies that included medication-naive patients and two studies that included antidepressant-naive patients, the depressed patients generally received antidepressants of different types and dosages with or without other types of medication. The majority of studies utilized the VFT as the active paradigm, while others adopted the verbal repetition task, hyperventilation and paper-bag breathing, visuospatial task, Stroop task, Sternberg's task, Tower of Hanoi, CO_2_ inhalation task, working memory (WM) task, word generation tasks (WGT), emotional Stroop task, facial emotion recognition task, image-recall task, mirror drawing task (MDT), trail-making test (TMT), or a combination of tasks [e.g., word fluency task (WFT) with right-finger-tapping task]. The fNIRS instruments that were used and the number of channels utilized, with a large proportion using the 52-channel ETG-4000, varied across studies. Most of the studies measured the concentration of oxy-Hb and deoxygenated hemoglobin (deoxy-Hb) during various tasks for distinguishing the depressed from the healthy. The probe is normally positioned at the frontal (usually prefrontal) and/or temporal areas, with the exception of two papers that measured the parietal area as well and one paper that measured only the left and right hemispheres in general. For the papers that included the assessment of the parietal brain areas, one of them included subjects suffering from Alzheimer's disease, and the parietal dysfunctions were linked to the initial phase of dementia (30). Other studies such as Rosenbaum et al. (72) examined the frontoparietal networks of patients with early- and late-life depression (LLD), which is associated with cognitive control. The study discovered that the frontoparietal networks appeared to be essential in LLD as patients suffering from LLD and memory impairment are shown to be at elevated risk for having dementia (75).

**Table 2:** Summary of fNIRS studies differentiating depressed patients from healthy controls.

Source	Country	Sample size (male/female)	Age (mean ± standard deviation)	Diagnostic criteria (instrument)	Psychopathology measure	Medication	NIRS device	Paradigm	Brain area	Main findings
Pu et al., (25)	Japan	MDD: 24 (12/12) HC: 26 (8/18)	MDD: 47.9 ± 13.9 HC: 42.4 ± 9.3	DSM-IV-TR (MINI)	BDI HAMD	All on antidepressants	52-Channel NIRS (ETG-4000)	2-Back task with blocked periodic baseline, activation	PF T	– MDD group had ↓ response sensitivity and accuracy than HC.
Matsuo et al. (26)	Japan	UNI: 8 (1/7) BP: 1 (1/0) HC: 10 (0/10)	UNI+BP: 65.6 ± 6.4 HC: 59.5 ± 5.9	DSM-IV (unspecified)	HAMD	All on medication	HEO-200	Verbal repetition task, VFT, Hyperventilation, Paper-bag breathing	F	– VFT: Oxy-Hb ↑ and deoxy-Hb ↓ in HC group but no noteworthy changes in depressed group. – Hyperventilation: oxy-Hb ↓ while deoxy-Hb ↑
Herrmann et al. (27)	Germany	MDD: 9 (5/4) HC: 9 (5/4)	MDD: 37.3 ± 13.8 HC: 35.1 ± 5.5	ICD-10	BDI	All on medication	2-Channel NIRO-300 monitor	VFT	PF	– Oxy-Hb ↑ in HC. – MDD had significantly ↓ activation.
Shoji et al. (28)	Japan	MDD: 26 HC: 32	NA	ICD-10	HAMD	NA	44-channel ETG-4000 (Hitachi)	Word generation tasks	PF	– Oxy-Hb variations in MDD were appreciably lesser than HC in all word tasks.
Kinoshita et al. (29)	Japan	MDD: 17	44.2 ± 12.2	DSM-IV (SCID)	HAMD	Majority on antidepressants	22-Channel ETG-4000 (Hitachi)	DEX/CRH test VFT	F	– Results did not fit well with the diagnostic criteria (DSM or ICD).
Kito et al. (30)	Japan	MDD: 30 (9/21) HC: 33 (11/22)	MDD: 71.1 ± 6.8 HC: 69.6 ± 5.5	DSM-IV	HAMD	All on medication	FOIRE-3000 (Shimadzu)	VFT	F, P	– Cortical activation in the VFT in MDD ↓ compared to HC.
Matsuo et al. (31)	Japan	MDD: 14 (4/10) HC: 21 (3/18)	MDD: 56.1 ± 17.3 HC: 50.3 ± 12.6	DSM-IV	HAMD	All on medication	Single channel HEO-200 (Omron)	VFT Hyperventilation Paper-bag breathing	F	– VFT: ↑ in oxy-Hb was lower in MDD compared to HC. – Hyperventilation: MDD demonstrated an appreciably smaller reduction in oxy-Hb than HC. – Paper-bag breathing: oxy-Hb ↑ in both groups while deoxy-Hb ↓ in MDD.
Matsubara et al. (32)	Japan	MDD: 10 HC: 10	NA	NA	NA	NA	52-Channel ETG-4000 (Hitachi)	Emotional Stroop task	T	– HC showed ↑ in oxy-Hb in the fronto-temporal regions as opposed to MDD.
Koike et al. (33)	Japan	MDD: 405 HC: 369	NA	NA	NA	NA	fNIRS	VFT	PF	– The intensity of signals was smaller in MDD. – The duration of time taken to complete the task was later in MDD.
Azechi et al. (34)	Japan	MDD: 30 HC: 30	NA	NA	NA	NA	2-Channel NIRS	VFT, TOH, SBT Stroop Task	F	– Task performances of the VFT and TOH were ↓ in MDD than in HC.
Matsuo et al. (35)	Japan	MDD: 10 (5/5) HC: 10 (6/4)	MDD: 62.2 ± 4.8 HC: 58.7 ± 5.8	DSM-IV (MINI)	HAMD	Only 4 patients had medication	24-Channel ETG-100 (Hitachi)	VFT WRT CO_2_ inhalation	PF	– During cognitive task, there was ↓ activation of PF cortex in MDD. – Negative association between ↓ PF activation while performing the cognitive task and degree of hyperintensity in the periventricular region or left F cortex in MDD. – CO_2_ inhalation causing vasomotor reactivity was ↓ in MDD than HC.
Ohta et al. (36)	Japan	MDD: 17 (5/12) HC: 24 (12/12)	MDD: 42.8 ± 18.2 HC: 36.2 ± 16.5	DSM-IV (MINI)	HAMD	All on medication	52-Channel ETG-4000 (Hitachi)	WFT	F	– Improvement of oxy-Hb values in the bilateral F cortices, – MDD showed attenuated ↑ in oxy-Hb while doing WFT in the bilateral F regions. – Hypofrontality in MDD is most notable in the left medial inferior F lobe. – Enhancement of oxy-Hb was not as notable on the right-sided channels in MDD.
Suto et al. (18)	Japan	MDD: 10 (9/1) HC: 16 (12/4)	MDD: 47.9 ± 12.8 HC: 42.9 ± 4.6	DSM-IV	HAMD	All on medication	24-Channel ETG-100 (Hitachi)	WFT Right-finger-tapping task	F, T	– During first half of task period, MDD had ↓ oxy-Hb increase than HC. ↑were seen in the anterior lower T and lower F channels. – Finger tapping tasks in MDD patients caused ↑ in oxy-Hb compared to HC.
Shimodera et al. (37)	Japan	UNI: 39 (19/20) BP: 14 (7/7) HC: 24 (13/11)	UNI: 56.9 ± 12.6 BP: 51.4 ± 14.0 HC: 40.9 ± 10.6	DSM-IV-TR	HAMD	All on medication	52-Channel OMM-3000/16 (Shimadzu)	VFT	PF	– Both UNI and BP showed ↓ area under curves (AUCs) than HC. – MDD showed significantly ↓ weighted center (WC) than BP or HC.
Ma et al. (38)	China	Menopausal MD: 30 (0/30) MDD: 30 (0/30) HC: 30 (0/30)	MD: 51.17 ± 6.06 MDD: 37.50 ± 10.60 HC: 34.83 ± 8.77	DSM-IV	HAMD	Only 20 patients took medication	45-Channel FOIRE-3000 (Shimadzu)	VFT	PF	– MD and MDD both showed ↓ oxy-Hb activation in bilateral DLPFC than HC, but involving different channels. – Atypical hemodynamics of the left DLPFC can discriminate HC from MDD using NIRS.
Takizawa et al. (39)	Japan	UNI: 153 (76/77) BP: 134 (65/69) HC: 590 (276/314)	UNI: 43.8 ± 12.7 BP: 44.0 ± 14.9 HC: 43.9 ± 15.7	DSM-IV SCID	HAMD	10 drug-free patients with UNI	52-Channel ETG-4000 (Hitachi)	VFT	PF, T	– NIRS can differentiate HC from patients, and differentiate UNI from BP.
Liske et al. (40)	Germany	MDD: 41 HC: 46	NA	NA	SIMS	NA	NA	Motor task–pressing button	P	– In HC, a noteworthy contrast in the NIRS signal resulting from the left P brain regions but this was weaker in MDD.
Zhu et al. (41)	China	UNI: 35 (11/24) BP: 39 (19/20) HC: 36 (18/18)	UNI: 35.9 ± 13.2 BP: 37.0 ± 12.9 HC: 33.6 ± 10.3	DSM-IV-TR (MINI)	HAMD	All on medication	52-Channel ETG-4000 (Hitachi)	1-Back version of the n-back WMT	PF, T	– Compared to HCs, UNI and BP ↓ activation of oxy-Hb in the inferior PF region during WMT. – Distinct prefrontal activation patterns in the Broca's area and left frontopolar region, underline BD and UD.
Matsubara et al. (42)	Japan	UNI: 16 (8/8) BP: 16 (8/8) HC: 20 (10/10)	UNI: 45.4 ± 12.2 BP: 44.1 ± 17.5 HC: 41.4 ± 8.5	DSM-IV-TR (MINI)	HAMD	All on medication	52-Channel ETG-4000 (Hitachi)	Emotional Stroop task	PF	-While doing the threat task, depressed patients revealed ↑ oxy-Hb in the left middle frontal region. – When performing happy task, depressed patients, did not display any meaningful changes in oxy-Hb.
Akashi et al. (43)	Japan	MDD: 52 (32/20) Subdivided into with/without discrepancy HC: 48 (21/27)	MDD: 41.8 ± 12.7 HC: 38.9 ± 9.5	DSM-IV (MINI)	Structured interview guide for HAMD (SIGH-D)	Most of the patients taking medications	52-Channel ETG-4000 (Hitachi)	VFT	F, T	– In the FT regions, upsurge in mean oxy-Hb were ↓ in MDD than in HC.
Gao et al. (44)	China	MDD: 27 (7/20) HC: 24 (11/13)	MDD: 40.78 ± 13.42 HC: 43.13 ± 11.28	DSM-IV	HAMD	All were medication free for at least 4 weeks	CW-NIRS	Facial emotion recognition	PF	– Hemodynamic variations between the bilateral PF cortex and left PF cortex may provide dependable predictors for diagnosing depression
Schecklmann et al. (45)	Germany	UNI: 16 (9/7) BP: 14 (3/11) HC: 15 (7/8)	UNI: 43.4 ± 9.8 BP: 40.8 ± 10.2 HC: 40.9 ± 8.0	ICD-10	BDI-II	All on medication	52-Channel ETG-4000 (Hitachi)	WMT	PF, F	Results discovered unspecific deficits that inhibited discrimination between bipolar and unipolar depression in domains of working memory.
Tomioka et al. ^3,4^ (46)	Japan	MDD: 25 (3/22) HC: 62 (14/48)	MDD: 51.9 ± 16.6 HC: 51.7 ± 17.2	DSM-IV	HAMD	Medication-naive	52-Channel ETG-4000 (Hitachi)	VFT	PF, T	– MDD showed ↓ oxy-Hb values when comparing to with HC in the bilateral F and T cortices at baseline. – Hypofrontality response to VFT may represent a potential trait marker for depression
Ohtani et al. ^3,4^ (47)	Japan	UNI: 10 (4/6) BP: 18 (9/9) HC: 14 (7/7)	UNI: 39.2 ± 12.1 BP: 39.7 ± 9 HC: 33.6 ± 8.3	DSM-IV-TR	HAMD	All on medication	52-Channel ETG-4000 (Hitachi)	VFT	PF, T	– UNI and BP showed ↓ activation than HCs in the bilateral ventrolateral PF cortex and the anterior part of the T cortex.
Masuda et al. ^3,4^ (48)	Japan	MDD: 47 Response group to SSRIs: 28 (15/13) Nonresponse group: 19 (6/13) HC: 63 (35/28)	Response group: 48.9 ± 2.9) Nonresponse group: 43.2 ± 3.3 HC: 41.7 ± 1.4	DSM-IV-TR	HAMD	Medication-naive	47-Channel ETG-7100 (Hitachi)	VFT	PF, T	– In FT region, hemodynamic responses were ↓ in patients with response and nonresponse than in HC prior to treatment. – In the medial F region, hemodynamic responses were ↑ in patients with response to prior treatment.
Feng et al. ^4^ (49)	China	MDD: 15 (7/8) HC: 15 (6/9)	MDD: 30.93 ± 13.47 HC: 30.87 ± 10.11	DSM-5 SCI	HAMD	NA	45-Channel FOIRE-3000 (Shimadzu)	VFT	PF	– After the music treatment, average active oxy-Hb values of some channels were ↑ in both HC and MDD. – After music therapy, patients with MDD demonstrated substantial activation in the OFC, DLPFC, and VMPFC.
Hirano et al. ^3,4^ (50)	Japan	MDD: 30 (11/19) HC: 108 (45/63)	MDD: 59.4 ± 14.2 HC: 58.9 ± 13	ICD-10	MADRS QIDS-SR	All on medication	52-Channel ETG-4000 (Hitachi)	VFT	PF, T	– MDD exhibited ↓ oxy-Hb values in the bilateral F cortex while doing VFT than HC.
Downey et al. ^4^ (51)	UK	MDD: 18 HC: 51	NA	NA	NA	NA	MiniNTS 4 detectors/24 sources (UCL)	VFT, N-back working memory tasks	PF	– MDD had ↓ bilateral PF cortex oxy-Hb responses to VFT unlike HC, and this was additionally ↓ after 4 ECT sessions. – On WM task, MDD showed PF cortex inhibition at baseline and a different time course of oxy– and deoxy-Hb following 4 ECT.
Rosenbaum et al. ^3^ (52)	Germany	MDD: 60 HC: 24	MDD: 40 ± 14.79 HC: 33 ± 11.45	DSM-IV (SCI)	PHQ-9 MADRS	32% of patients treated with antidepressant medication	52-Channel ETG-4000 (Hitachi)	7-min resting phase, VAS, rumination response scale	P	– MDD as compared to HC, showed ↓ functional connectivity in parts of the DMN.
Kondo et al. ^3^ (53)	Japan	MDD: 25 (17/8) HC: 25 (18/7)	MDD: 36 ± 8.91 HC: 34.1 ± 10.1	DSM-IV-TR SCI	HAMD	All medicated with antidepressants	44-Channel ETG-4000 (Hitachi)	Image-recall task	F, T	– The oxy-Hb in HC was ↑ compared to MDD in bilateral F region. – The severity of depression was related to ↓ in oxy-Hb in left F lobe.
Zhu et al. ^3^ (54)	China	Affective disorder (AD): 28 (8/20) HC: 30 (21/9)	AD: 23.32 ± 5.01 HC: 23.60 ± 2.03	DSM-IV	SDS	13 patients were medicated. 15 free of any medicine	42-Channel FOIRE-3000 (Shimadzu)	Resting state measurements	F	– Relative to HC, AD demonstrated ↓ in intraregional and symmetrical interhemispheric connectivity in the PFC, revealed ↓ locally functional connectivity in the right IFG, and ↓ long-distance connectivity concerning the bilateral IFG.
Okada et al. ^3^ (55)	Japan	MDD: 36 (24/12) HC: 36 (21/12)	MDD (male): 23.3 ± 2.5 MDD (female): 21.3 ± 1.1 HC (male): 23.9 ± 2.4 HC (female): 23.6 ± 2.1	DSM-III-R	HAMD	13 patients received antidepressant medication. 23 were medication free for a minimum of 3 months	Multichannel near-IR spectrophotometry (NIRS)	MDT	LT, RT brain	– Nearly half of patients revealed a “nondominant hemisphere response pattern” – The supposedly “nondominant” hemisphere may convert to being dominant during the depression
Uemura et al. ^3^ (56)	Japan	MDD: 13 (6/7) HC: 67 (28/39)	MDD: 74.5 ± 5.8 HC: 73.8 ± 5.3	NA	GDS	All subjects were medicated.	8-Channel FOIRE– 3000; (Shimadzu)	Trail-making test, part B (TMT-B; tablet version)	PF	– Oxy-Hb activation when performing the TMT-B was ↓ in MDD in both the right and left PF cortex. – ↓ PF activation in the elderly with depressive symptomology may cause deterioration in executive function.
Kinou et al.^3^ (57)	Japan	MDD: 32 (15/17) HC: 32 (15/17)	MDD: 44.8 ± 9.8 HC: 45.7 ± 13.5	DSM-IV	H	All, except 3 patients	52-Channel ETG-4000 (Hitachi)	VFT	PF	– MDD revealed ↓ oxy-Hb activation during the task.
Yamagata et al.^3^ (58)	Japan	Early-onset depression (EOD): 11 (2/9) Late-onset (LOD): 12 (3/9) HC: 13 (8/5)	EOD: 68.4 ± 5.6 LOD: 70.2 ± 1.9 HC: 70.3 ± 4.4	DSM-IV	HAMD	All patients were taking one prescribed antidepressant.	52-Channel ETG-4000 (Hitachi)	WFT	F, T	– HC demonstrated ↑ in oxy-Hb vs. surges in oxy-Hb being minimally ↓ in EOD and extremely ↓ in LOD, bilaterally throughout the F cortices and T areas. – Attenuated activation in the left lateral PF and T areas may help to differentiate LOD and EOD.
Noda et al.^3^ (59)	Japan	MDD: 30 (14/16) HC: 30 (14/16)	MDD: 36.7 ± 11.6 HC: 35.1 ± 9.4	DSM-IV (SCI)	GRID-HAMD	All patients medicated with antidepressants.	52-Channels ETG-4000 (Hitachi)	VFT	F. T	– Oxy-Hb increases while performing task was meaningfully ↓ in MDD. – ↓ Right F-T activation on NIRS during VFT is related to the MDD severity.
Nishizawa et al. (60)	Japan	MDD: 14 (7/7) HC: 20 (13/7)	MDD: 38.2 ± 12.9 HC: 29.0 ± 5.7	DSM-IV-TR (SCID)	HAMD	All taking antidepressants except for four patients.	22-Channel ETG-4000 (Hitachi)	Emotional Stroop task	F, T	– Hyperactivated oxy-Hb was witnessed in the left F cortex on contact to unfavorable stimuli, but no noteworthy dissimilarity was established between MDD and HC on exposure to favorable stimuli.
Akiyama et al. ^3^ (61)	Japan	MDD: 177 (73/104) HC: 50 (40/10)	MDD: 47.2 ± 15.1 HC: 32.7 ± 7.5	DSM-IV-TR	HAMD	All patients were on antidepressants	52-Channel ETG-4000 (Hitachi)	VFT	PF, T	– Significant hypoactivation in bilateral F-T regions was observed in MDD compared with HC.
Ohi et al.^3^ (62)	Japan	UNI: 26 (17/9) BP: 22 (13/9) HC: 51 (33/18)	UNI: 41.1 ± 12.7 BP: 39.9 ± 12.5 HC: 35.7 ± 11.9	DSM-V	HAMD	Chlorpromazine equivalents of total antipsychotics	52-Channel ETG-4000 (Hitachi)	VFT	PF	– UNI and BP groups had ↓ PF activity than HC. – Patients with and without family history had ↓ PF activity than HC subjects. – Demonstrate connection of more serious PF dysfunction with higher genetic loading for disease.
Takei et al.^3^ (63)	Japan	UNI: 29 (14/15) BP: 31 (14/17) HC: 31 (11/20)	UNI: 34.5 ± 9.0 BP: 34.9 ± 6.6 HC: 33.6 ± 10.0	DSM-IV	HAMD	Nearly all patients were on medication.	52-Channel ETG-4000 (Hitachi)	Conversation task and control task	F, T	– Both UNI and BP showed ↓ of continuous activation in the left DLPFC and left FPC, and decreased rapid change in bilateral FPC activation. – F activation while conversing ↓ in equally in UNI and BP. – Pathophysiological character of UNI and BP are reflected in continuous activation and rapid change change Impaired adaptive ability in UNI may be related to ↓ amount of rapid change in right FPC.
Pu et al.^3^ (64)	Japan	Late-onset depression (LOD): 24 (6/18) HC: 30 (14/16)	LOD: 72.3 ± 5.5 HC: 72.0 ± 4.7	DSM-IV (MINI)	BDI HAMD	Antidepressant-naive	52-Channel ETG-4000 (Hitachi)	VFT	PF, T	– LOD had ↓ activation in both PF and superior T cortices than HC. – ↓ frontopolar cortical activation was linked with social functioning impairment in LOD.
Tsujii et al.^3^ (65)	Japan	MDD: Suicide attempters (SAs): 30 (8/22) Nonattempters (NAs): 38 (16/22) HC: 40 (15/25)	MDD (SAs): 37.6 ± 10.0 MDD (NAs): 38.8 ± 9.7 HC: 38.2 ± 10.5	DSM-IV (MINI)	HAMD	All on medication.	52-Channel ETG-4000 (Hitachi)	VFT	F, T	– MDD had significantly ↓ activation in the bilateral FT regions compared to HCs. – SAs demonstrated ↓ hemodynamic response in the left precentral gyrus than HCs and NAs. – Aggression and hopelessness were negatively associated with hemodynamic responses in the right middle F gyrus in SAs but not in HCs and NAs.
Pu et al.^3^ (66)	Japan	Late-onset depression (LOD): 36 (9/27) HC: 35 (11/24)	LOD: 71.8 ± 5.1 HC: 70.9 ± 4.3	DSM-IV (MINI)	BDI HAMD	Antidepressant-naive	52-Channel ETG-4000 (Hitachi)	Working memory (WM) task	PF, T	– LOD was correlated with ↓ PF and T activation when comparing with HC. – Hemodynamic response in PF and T regions when performing WM task could correlate with social functioning in LOD.
Tsujii et al.^3^ (67)	Japan	MDD with melancholia (MDD-MF): 30 (15/15) MDD without -melancholia (MDD-NMF): 52 (18/34) HC: 68 (32/36)	MDD-MF: 42.2 ± 11.8 MDD-NMF: 40.6 ± 11.7 HC: 40.5 ± 10.6	DSM-IV (MINI)	HAMD	All on medication	52-Channel ETG-4000 (Hitachi)	VFT	F, T	– Both MDD groups demonstrated ↓ hemodynamic responses in the F-T regions.
Liu et al.^3^ (68)	China	MDD: 30 (12/18) HC: 30 (16/14)	MDD: 38.38 ± 12.8 HC: 33.2 ± 10.5	DSM-IV-TR	HAMD	Free of medication	52-Channel FOIRE-3000 (Shimadzu)	VFT	PF	– ↓ Activation in lateral and lower PFC in MDD. – Antero-medial PFC and bilateral PFC were correlated with the degree of depressive symptoms. – MDD patients with obsession–compulsion symptoms and anxiety exhibited a PFC ↓ activation state in NIRS.
Wang et al.^3^ (69)	China	First-episode MDD (fMDD): 36 (15/21) Recurrent MDD: 34 (11/23) HC: 37 (22/15)	fMDD: 38.75 ± 13.86 Recurrent MDD: 43.26 ± 13.85 HC: 35.70 ± 11.39	DSM-IV	HAMD	All were on antidepressants	52-Channel ETG-4000 (Hitachi)	VFT	F, T	– In comparison with HC and fMDD, chronic MDD had significantly ↓ brain activation over right PF and superior T cortices. – Variation in activations in bilateral F and T regions. HC: more channels in left than right hemisphere; fMDD: more channels in right than left hemisphere; recurrent MDD: only channels in left hemisphere.
Tsujii et al.^3^ (70)	Japan	MDD with melancholia (MDD-MF): 32 (16/16) MDD without melancholia (MDD-NMF): 28 (15/13) HC: 24 (11/13)	MDD-MF: 40.8 ± 15.3 MDD-NMF: 38.9 ± 11.8 HC: 38.6 ± 9.2	DSM-IV (MINI)	SIGH-D BDI-II	All on medication	52-Channel ETG-4000 (Hitachi)	VFT	F, T	– Noteworthy disparities were witnessed in mean oxy-Hb fluctuations of MDD-MF in 25 channels and in those with MDD-NMF in 12 channels compared to HC.
Nishida et al.^3^ (71)	Japan	MDD: 14 (7/7) HC: 15 (8/7)	MDD: 46.2 ± 11.9 HC: 45.5 ± 10.9	DSM-IV-TR (MINI)	HAMD	All on medication	52-Channel ETG-4000 (Hitachi)	VFT	PF, T	– MDD revealed a ↓ oxy-Hb activation than HC, predominantly in ventrolateral PF and T cortex regions.
Rosenbaum et al.^3^ (72)	Germany	Depressed: 49 Nondepressed: 51 2 participants diagnosed with bipolar disorder and eating disorder.	Depressed: 64.08 ± 7.06 Nondepressed: 64.16 ± 6.14	NA	BDI GDS	54% took medication.	38-Channel ETG-4000 (Hitachi)	Adapted trail-making test (TMT-A, TMT-B, and TMT-C)	F, P	– Depressed and nondepressed revealed substantial differences in functional connectivity (FC) while doing the task performance and at rest. – During task performance, depressed patients exhibited ↓ FC in a left frontopolar cortical network, and ↑ FC in a left frontoparietal cortical network at the resting state and altered FC and network organization during different mental states.
Pu et al.^3^ (73)	Japan	MDD: 67 (29/38) MDD with suicidal ideation: 31 (11/20) MDD without suicidal ideation: 36 (18/18) HC: 67 (29/38)	MDD: 58.1 ± 16.0 MDD with suicidal ideation: 57.3 ± 15.7 MDD without suicidal ideation: 58.7 ± 16.5 HC: 58.1 ± 17.8	DSM-IV (MINI)	HAMD	All on antidepressants	52-Channel ETG-4000 (Hitachi)	VFT	PF, T	– Regional hemodynamic changes were considerably ↓ in MDD than in HCs in PF and T regions.
Pu et al.^3^ (74)	Japan	MDD: 26 (11/15) HC: 30 (12/18)	MDD: 47.9 ± 19.2 HC: 50.5 ± 19.7	DSM-IV-TR (MINI)	BDI HAMD	All on antidepressants	52-Channel ETG-4000 (Hitachi)	VFT	PF, T	– Regional hemodynamic changes were appreciably ↓ in MDD than in HC in PF and T regions, and was correlated positively with task-oriented coping (adaptive coping) in the bilateral ventrolateral and dorsolateral prefrontal cortex, and the midline frontopolar and bilateral orbitofrontal cortex regions.

^3^This article can also be found in the summary in **Table 3**.

^4^This article can also be found in the summary in **Table 4**.

MDD, major depressive disorder; HC, healthy control; BDI, Beck depression inventory; HAMD, Hamilton depression rating scale; UNI, unipolar depression; BP, bipolar disorder; VFT, verbal fluency task; PF, prefrontal; T, temporal, F, frontal; NA, not available; TOH, Tower of Hanoi; SBT, Sternberg's task; WRT, word repetition task; MINI, Mini-international neuropsychiatric interview; DSM, Diagnostic and Statistical Manual; ICD, International Classification of Disease; SCID, structured clinical interview for DSM-IV; SIMS, structured inventory of malingered symptomatology; WMT, working memory task; IFG, inferior frontal gyrus; MDT, mirror drawing task; DLPFC, dorsolateral prefrontal cortex; PFC, prefrontal cortex; SIGH-D, structured interview guide for the Hamilton depression rating scale.

The overall results showed that the depressed patients showed a smaller oxy-Hb increase than the controls, with a smaller increase in the frontal (especially the prefrontal) and temporal activation (appreciably lower activation in the depressed compared to HCs) while performing the WM task, VFT, WGT, and TMT. Kito et al. (30) measured both the frontal and parietal brain areas, and the results showed decreased cortical activation throughout the VFT in patients with depression and the HCs. With the same brain areas being measured, the depressed patients and HCs showed noteworthy dissimilarities in functional connectivity (FC) both in the resting state and during TMT performance, with depressed patients showing a decrease, and HCs showing a surge, in FC from the resting state to TMT performance (72). When subjects were asked to hyperventilate, there was a significant decrease in oxy-Hb levels, and deoxy-Hb was considerably elevated in the depressed and HCs, with the depressed patients showing a lesser reduction in oxy-Hb than healthy individuals (26, 31). During paper-bag breathing, Matsuo et al. (31) revealed that oxy-Hb significantly increased in both groups, whereas deoxy-Hb significantly decreased in the depressed juxtaposed to the healthy. Matsuo et al. (26) established no noteworthy alterations in oxy-Hb and deoxy-Hb in depressed patients or in HCs. Using the emotional Stroop task, Matsubara et al. found that the HCs compared to the depressed patients showed notably increased oxy-Hb during the happy-word trials, but the decreases in oxy-Hb during the threat-word trials were comparable in both depressed patients and HCs (32). However, Matsubara et al. and Nishizawa et al. concluded that the depressed when comparing with HCs showed notable increased in oxy-Hb within the left middle frontal region while performing the threat task, whereas depressed patients compared to HCs showed no noteworthy variation in oxy-Hb while performing the happy task (42, 60). For the CO_2_ inhalation task, the vasomotor reactivity was significantly decreased in the depressed patients than controls (35). When subjects were performing right-finger-tapping tasks, the increase in oxy-Hb was higher in the depressed patients when comparing with HCs (18). In the image-recall task, the change in oxy-Hb in the HCs was considerably more than in the depressed while experiencing unpleasant conditions (53).

Almost all of the studies did not indicate the specificity and sensitivity of differentiating between depressed patients and HCs, with the exception of one that indicated an 80% sensitivity but with its specificity being unspecified (29). One study had indicated a sensitivity of 71.5% and a specificity of 70% for differentiating depressed patients from those with Alzheimer's disease (30), and another study mentioned a sensitivity of 0.71 and a specificity of 0.46 to distinguish between the euthymic, unipolar and bipolar depressive patients (37). Last, there was one study that indicated different sensitivities and specificities, which were dependent on the integral values of the two regions of interest (region 1 consisted of frontopolar and dorsolateral prefrontal cortical regions, while region 2 consisted of the middle and superior temporal cortical regions and the ventrolateral prefrontal cortex) (39).

### Correlation of fNIRS Signals With Depression Symptomatology

We identified 31 eligible papers reporting on fNIRS studies in which cerebral hemodynamic changes were correlated with depression symptomatology in a total of 1424 patients (**Table 3**). These studies were generally in those with MDD, but some were inclusive of patients with other mental health illness, for example: bipolar disorder, affective disorder, and schizophrenia. The majority of the studies adopted the VFT, except for 12 studies that utilized paradigms such as WFT, RPS task, image-recall task, resting state measurements, TMT, stop-signal task, WM task, MDT, conversation task, emotional Stroop task, and the use of self-report measures such as the rumination response scale (RRS) and visual analog scales (VAS). The NIRS instruments utilized markedly differed from study to study.

**Table 3:** Summary of fNIRS studies correlating cerebral hemodynamic changes with depression symptomatology.

Source	Country	Sample size (male/female)	Age (mean ± standard deviation)	Diagnostic criteria instrument	Psychopathology measure for symptomatology	Specific symptomatology	NIRS device/no. of channels	Paradigm	Brain area	Main finding
Kawano et al. (76)	Japan	N: 25 *included other disorders	44.1 ± 9.3	DSM-IV	HAMD	Depressive symptoms	22-Channel ETG-4000 (Hitachi)	VFT	F, T	– The integral value of blood flow in frontal lobe was negatively correlated with the degree of depression
Onishi et al.^4^ (77)	Japan	N: 10 (5/5)	71.0 ± 6.0	DSM-IV	HAMD MMSE	Depressive symptoms Cognitive functioning	48-Channel ETG-4000 (Hitachi)	Rock, paper, scissors (RPS)	PF	– Negative correlation between ratio of HAMD and oxy-Hb was observed.
Hirano et al.^2,4^ (50)	Japan	N: 30 (11/19) *inclusive of bipolar patients	59.4 ± 14.2	ICD-10	MADRS QIDS-SR MMSE	Depressive symptoms Cognitive functioning	52-Channel ETG-4000 (Hitachi)	VFT	PF, T	– After ECT, the reduction of degree of depression was associated with increase in oxy-Hb values in the right ventrolateral PF cortex. – Changes in oxy-Hb is significantly interrelated with MADRS score ↓ but not significantly correlated with ↓ in total QIDS-SR total scores.
Masuda et al.^2,4^ (48)	Japan	N: 47 Response group to SSRIs: 28 (15/13) Nonresponse group: 19 (6/13)	Response group: 48.9 ± 2.9 Nonresponse group: 43.2 ± 3.3	DSM-IV	POMS STAI DACS	Anxiety symptoms Depressive symptoms	47-Channel ETG-7100 (Hitachi)	VFT	PF, T	– Hemodynamic responses in medial F region were significantly greater before treatment in patients with a response to SSRIs than those with no response.
Kondo et al.^2^ (53)	Japan	N: 25 (17/8)	36 ± 8.91	DSM-IV	HAMD	Depressive symptoms	44-Channel ETG-4000 (Hitachi)	Image-recall task	F, T	– A noteworthy negative correlation between oxy-Hb and HAMD score was seen in left F region during the unpleasant condition. – ↓ in oxy-Hb in left F lobe was associated to degree of depression.
Koseki et al. (78)	Japan	MDD: 75 (39/36)	39.23 ± 12.49	DSM-IV	HAMD ATQ-R NART STAI	Depressive symptoms Automatic thoughts State/trait anxiety	52-Channel ETG-4000 (Hitachi)	VFT	PF, T	– Activation in right superior temporal gyrus was associated to deviation to negative of the proportion of negative and positive thought.
Zhu et al.^2^ (54)	China	Affective disorder (AD): 28 (8/20)	23.32 ± 5.01	DSM-IV	SDS	Depressive symptoms	42-Channel FOIRE-3000 (Shimadzu)	Resting state	F	– Degree of self-reported symptoms of depression was negatively associated with strength of intraregional and symmetrically interhemispheric connectivity in the PFC.
Uemura et al.^2^ (56)	Japan	N: 13 (6/7)	74.5 ± 5.8	NA	GDS MMSE	Depressive symptoms	8-Channel FOIRE-3000 (Shimadzu)	TMT-B	PF	– Both oxy-Hb activation in left and right hemisphere were significantly negatively associated with GDS.
Noda et al.^2^ (59)	Japan	N: 30 (14/16)	36.7 ± 11.6	DSM-IV (SCID-I)	GRID-HAMD	Depressive symptoms	52-Channels ETG-4000 (Hitachi)	VFT	F, T	– Average increase in oxy-Hb during the task revealed a significant negative association with the HAMD total scores.
Akiyama et al.^2^ (61)	Japan	N: 177 (73/104)	47.2 ± 15.1	DSM-IV-TR	HAMD PHQ-9	Depressive symptoms	52-Channel ETG-4000 (Hitachi)	VFT	PF, T	– Left lateral F-T activation was significantly ↓ in the group with depressed mood or anhedonia.
Tomioka et al.^2,4^ (46)	Japan	N: 25 (3/22)	51.9 ± 16.6	DSM-IV	HAMD	Depressive symptoms	52-Channel ETG-4000 (Hitachi)	VFT	PF, T	-Patients with MDD who revealed ↑ baseline oxy-Hb activation while performing VFT in the inferior F and middle T regions showed more improvements in depressive symptoms after being treated.
Rosenbaum et al.^2^ (72)	Germany	N: 49 *inclusive of other disorders	64.08 ± 7.06	NA	BDI GDS	Depressive symptoms Cognitive functioning	38-Channel ETG-4000 (Hitachi)	TMT-A TMT-B TMT-C	F, P	– Depressed patients revealed ↓ FC in a left frontopolar cortical network while doing task performance ↑ FC in a left frontoparietal cortical network at the resting state.
Yamagata et al.^2^ (58)	Japan	Early-onset depression (EOD): 11 (2/9) Late-onset depression (LOD): 12 (3/9)	EOD: 68.4 ± 5.6 LOD: 70.2 ± 1.9	DSM-IV	HAMD MMSE	Depressive symptoms	52-Channel ETG-4000, NIRS system (Hitachi)	WFT	F, T	– HAMD score exhibited negative correlations with oxy-Hb for two channels.
Akashi et al. (79)	Japan	n = 48	NA	NA	NA	Inhibitory deficit	47-Channel NIRS	Stop-signal task	F	– MDD showed the variations of brain dysfunctions correlated in the inhibitory controls.
Satomura et al. (80)	Japan	Initial (T0)–N: 65 After 1.5 years (T1.5)–N: 45	39.8 ± 11.8	DSM-IV (SCID-I)	HAMD GAF	Depressive symptoms Global functioning	52-Channel ETG-4000 (Hitachi)	VFT	PF, T	– Brain activation in the bilateral MFG and right IFG as calculated by NIRS may differentially denote clinical severity and trait-related anomalies in MDD.
Ohtani et al.^2,4^ (47)	Japan	N: 10 (4/6)	39.2 ± 12.1	DSM-IV	SASS HAMD	Social adaptation Depressive symptoms	52-Channel ETG-4000 (Hitachi)	VFT	PF, T	– Longitudinal changes in SASS scores were positively related with magnitude of change in the right VLPFC/aTC activation in MDD group.
Pu et al.^2^ (64)	Japan	Late-onset MDD (LOD): N: 24 (6/18)	72.3 ± 5.5	DSM-IV (MINI)	BDI HAMD SASS MMSE	Depressive symptoms Social functioning	52-Channel ETG-4000 (Hitachi)	VFT	PF, T	– Average oxy-Hb changes in LOD patients had a significantly positive association with SASS scores.
Pu et al.^2^ (66)	Japan	Late-onset depression (LOD): 36 (9/27)	71.8 ± 5.1	DSM-IV (MINI)	BDI HAMD SASS MMSE	Depressive symptoms Social functioning	52-Channel ETG-4000 (Hitachi)	Working memory (WM) task	PF, T	– Reduced activation in PF and T regions was significantly associated to ↓ scores on the SASS in patient group and might act as a biological marker of social functioning in LOD patients.
Pu et al.^2^ (74)	Japan	N: 26 (11/15)	47.9 ± 19.2	DSM-IV-TR (MINI)	HAMD BDI CISS	Depressive symptoms Coping styles	52-Channel ETG-4000 (Hitachi)	VFT	PF, T	– Regional hemodynamic changes were ↓ in MDD group compared to the control group in PF and T areas, and positively interrelated with task-oriented coping (adaptive coping) in the various PF regions.
Pu et al.^2^ (81)	Japan	N: 67, 31 with suicidal ideation and 36 without	58.1 ± 16.0	DSM-IV (MINI)	HAMD	Suicidal ideation	52-Channel ETG-4000 (Hitachi)	VFT	PF, T	– Hemodynamic variations correlated negatively with the degree of suicidal thinking in OFC, FPC, and DLPFC.
Nishida et al.^2^ (71)	Japan	N: 14 (7/7)	46.2 ± 11.9	DSM-IV-TR (MINI)	HAMD PSQI ESS	Depressive symptoms Sleep quality	52-Channel ETG-4000 (Hitachi)	VFT	PF, T	– Significant negative correlations between average oxy-Hb variations during VFT and PSQI scores. – Mean oxy-Hb changes showed no significant correlations with ESS scores. – No significant association between average oxy-Hb variations during the task and sleep variables.
Tsujii et al.^2^ (70)	Japan	MDD with melancholia (MDD-M): 32 (16/16) MDD without melancholia (MDD-NM): 28 (15/13)	MDD-MF: 40.8 ± 15.3 MDD-NMF: 38.9 ± 11.8	DSM-IV (MINI)	BDI-II SIGH-D GAF	Depressive symptoms Psychomotor retardation	52-Channel ETG-4000 (Hitachi)	VFT	F, T	– No meaningful associations between average oxy-Hb changes and GAF/HAMD/BDI-II total scores. – Psychomotor retardation on HAMD indicated noteworthy positive correlation with average oxy-Hb changes in right T areas in MDD-MF but revealed significant negative association with average oxy-Hb changes in the middle to left T region in MDD-NMF.
Tsujii et al.^2^ (67)	Japan	MDD with melancholia (MDD-M): 30 (15/15) MDD without melancholia (MDD-NM): 52 (18/34)	MDD-M: 42.2 ± 11.8 MDD-NM: 40.6 ± 11.7	DSM-IV (MINI)	HAMD SF-36	Depressive symptoms Quality of life	52-Channel ETG-4000 (Hitachi)	VFT	F, T	MDD-M patients reveal qualitatively dissimilar prefrontal dysfunction patterns correlated with emotional role functioning as compared to MDD-NM patients.
Wang et al.^2^ (69)	China	First-episode MDD (fMDD): 36 (15/21) Recurrent MDD (rMDD): 34 (11/23)	fMDD: 38.75 ± 13.86 rMDD: 43.26 ± 13.85	DSM-IV	HAMD	Depressive symptoms	52-Channel ETG-4000 (Hitachi)	VFT	F, T	-rMDD group had ↓ increases in oxy-H comparing to the fMDD group.
Liu et al.^2^ (68)	China	N: 30 (12/18)	38.38 ± 12.8	DSM-IV-TR	HAMD HAMA Y-BOCS	Depressive, anxiety, obsessive–compulsive symptoms	52-Channel FOIRE-3000 (Shimadzu)	VFT	PF	– Average oxy-Hb changes revealed significant positive correlation with HAMD scores. -No statistical relationship was witnessed on the degree of obsessive–compulsive symptoms.
Rosenbaum et al.^2^ (52)	Germany	N: 60	40 ± 14.79	DSM-IV (SCI)	PHQ-9 MADRS	Depressive symptom State and trait aspect of rumination	52-Channel ETG-4000 (Hitachi)	RRS VAS Self-report on inner experience	P	– Subjects who are depressed revealed ↓ functional connectivity in parts of the DMN compared to HCs. – mind-wandering revealed positive associations, whereas rumination was negatively associated with FC in the cortical parts of the DMN
Okada et al.^2^ (55)	Japan	N: 36 (24/12)	Male: 23.3 ± 2.5 Female: 21.3 ± 1.1	DSM-III-R	HAMD	Depressive symptoms	Multichannel near-IR spectrophotometry	Mirror drawing task (MDT)	Left and right hemispheres	– Nearly half of the patients revealed a “nondominant hemisphere response pattern,” which was not witnessed in normal subjects during the MDT. – During the course of depression, the supposedly “nondominant” hemisphere may become dominant.
Kinou et al.^2^ (57)	Japan	N: 32 (15/17) *included HC, MDD, schizophrenia	44.8 ± 9.8	DSM-IV	HAMD Global assessment of functioning	Depressive symptoms	52-Channel ETG-4000 (Hitachi)	VFT	PF	– Lower Global Functioning scores were correlated with ↓ hemodynamic responses.
Takei et al.^2^ (63)	Japan	N: 29 (14/15)	34.5 ± 9.0	DSM-IV	HAMD GAF	Depressive symptoms Cognitive functioning	52-Channel ETG-4000 (Hitachi)	Conversation task and control task	F, T	-Prompt change in activation was positively associated with GAF scores in the MDD patients.
Ohi et al.^2^ (62)	Japan	N: 26 (17/9)	41.1 ± 12.7	DSM-5	HAMD Clinical interview for family history	Depressive symptoms Family history/familial loadings	52-Channel ETG-4000 (Hitachi)	VFT	PF	– Illustration of the association of significantly more severe PF dysfunction with higher genetic loading in major mental illnesses.
Tsujii et al.^2^ (65)	Japan	MDD: suicide attempters (SAs): 30 (8/22) nonattempters (NAs): 38 (16/22)	MDD (SAs): 37.6 ± 10.0 MDD (NAs): 38.8 ± 9.7	DSM-IV (MINI)	HAMD Barratt impulsiveness scale Buss– Perry aggression questionnaire Beck hopelessness scale	Depressive symptoms Impulsivity Aggression Hopelessness	52-Channel ETG-4000 (Hitachi)	VFT	F, T	– SAs revealed smaller hemodynamic response in the left precentral gyrus than NAs and HCs. – Hemodynamic responses in the right middle F gyrus were negatively correlated with aggression and hopelessness in SAs.

^2^This article can also be found in the summary in **Table 2**.

^4^This article can also be found in the summary in **Table 4**.

MDD, major depressive disorder; HC, healthy control; HAMD, Hamilton depression rating scale; VFT, verbal fluency task; PF, prefrontal; T, temporal, F, frontal; NA, not available; MINI, Mini-international neuropsychiatric interview; DSM, Diagnostic and Statistical Manual; ICD, International Classification of Disease; MMSE, Mini-Mental State Examination; SASS, school and staffing survey; POMS, profile of mood states; STAI, state-trait anxiety inventory; MADRS, Montgomery–Asberg depression rating scale; QIDS-SR, quick inventory of depressive symptomatology-self report; DSRS, dementia severity rating scale; HAMA, Hamilton anxiety rating scale; GDS, geriatric depression scale; PHQ, patient health questionnaire; BDI, Beck depression inventory; TMT, trail-making test; SCID, structured clinical interview for DSM-IV; IFG, inferior frontal gyrus; MFG, medial frontal gyrus; DLPFC, dorsolateral prefrontal cortex; OFC, orbitofrontal cortex; FPC, frontopolar cortex; PSQI, Pittsburgh sleep quality index; ESS, Epworth sleepiness scale; SIGH-D, structured interview guide for the Hamilton depression rating scale; Y-BOCS, Yale–Brown obsessive–compulsive scale; RRS, rumination response scale; VAS, visual analog scale; DMN, default mode network; MDT, mirror drawing task; GAF, global assessment of functioning; ATQR, Automatic thoughts questionnaire-revised.

According to our assessment of the authors' conclusions, almost every study discovered that oxy-Hb concentration values were negatively correlated with the degree of total depressive symptoms [HAMD, geriatric depression scale (GDS), Montgomery–Asberg depression rating scale (MADRS), quick inventory of depressive symptomatology-self rated (QIDS-SR), etc.] and Uemura et al. even demonstrated that such a significant negative correlation was still present after correcting for confounding factors such as gender, age, and educational history (56). Such a negative correlation was further supported by Wang et al. who illustrated that the recurrent MDD group had notably diminished increases in oxy-Hb compared to first-episode MDD patients (69). Additionally, Nishizawa et al. utilized the emotional Stroop task to evaluate brain regions associated with the severity of depression. They found that hyperactivated oxy-Hb was seen in the left frontal cortex upon contact to adverse stimuli, but no meaningful differences were established between the depressed compared with the healthy upon exposure to favorable stimuli (60). Additionally, the degree of depression was contrariwise associated with an evoked wave in the left upper frontal cortex following exposure to favorable stimuli. Akiyama et al. also found that there was significant decrease in left lateral frontotemporal activation in active depressed patients as compared to those who still had residual depressive symptoms despite MDD remission (61), further suggesting the possibility of NIRS's ability to differentiate depression of varying severity.

While such a noteworthy negative correlation was established in many papers, there were some studies that observed no significant correlations (70) and even a significant positive association (68) between HAMD scores and oxy-Hb concentrations. Such results could have been due to methodological limitations that were faced by these studies. For example, in the study by Tsujii et al. (70), the sample size was small. Thus, such slight difference in the hemodynamic response in the left temporal region among the two groups was not statistically different. A type II error might be a possibility if the size of the sample was enlarged, and the dissimilarity between the healthy and depressed in the left channels might be meaningful. Additionally, Liu et al. (68) argued that such inconsistency in results could have been due to the dynamic process of psychiatric disease development; therefore, there are some findings that may be momentary markers and not persistent markers.

Apart from the general negative association between the oxy-Hb concentration values and the degree of total depressive symptoms, many of the studies looked into a variety of individual depressive symptoms, such as suicidal thoughts, sleep quality, psychomotor retardation, obsessive–compulsive symptoms, cognitive functioning [assessed by the Mini-Mental State Examination (MMSE), Consortium to Establish a Registry for Alzheimer's Disease (CERAD) test battery, etc.], and many others.

Suicidality is often observed in MDD patients. Suicidal ideation usually precedes a suicide attempt. Pu et al. investigated individuals prior to any attempt (73), while Tsujii et al. explored responses after an attempt (65). Pu et al. (73) found that the hemodynamic variations in the OFC, FPC, and DLPFC negatively correlated to the degree of suicidal thinking in depressed individuals; Tsujii et al. (65) revealed that the suicide attempters (SAs) revealed a reduced hemodynamic response in the left precentral gyrus compared to the nonattempters (NAs) and HCs, and the hemodynamic responses in the right middle frontal gyrus were negatively correlated with aggression and hopelessness in the SAs but not in the HCs and NAs.

Nishida et al. examined the relationship between sleep quality and oxy-Hb concentrations (71). They detected a negative relationship between average oxy-Hb concentration variations while performing the task and Pittsburgh sleep quality index (PSQI) scores. This illustrated that self-rated sleep disturbances were related to a reduced left prefrontal reactivity when performing VFT in MDD patients, thus indicating that the reactivity of the prefrontal region was vulnerable to sleep disorders.

Psychomotor retardation is another classic symptom of MDD. In Tsujii et al., psychomotor retardation on the HAMD showed noteworthy affirmative association with average oxy-Hb changes in the right temporal region in the MDD patients with melancholic features (MDD-MF) but displayed a suggestive negative association with average oxy-Hb changes in the middle to left temporal region in the MDD patients with non-melancholic features (MDD-NMF) (70). These findings were consistent with earlier functional neuroimaging studies that determined that MDD with psychomotor retardation is related to reduced blood flow in patients with MDD-NMF, unlike those suffering from MDD-MF. Thus, this suggested that the pathophysiology of non-melancholic MDD is distinctive from melancholia. Specifically, the pathophysiology of melancholia could be linked to the right temporal region. MDD patients often showed impaired inhibitory control. In the study by Akashi et al. fluctuations in oxy-Hb concentrations in MDD patients in the superior frontal probes were associated with impairments in performance in inhibitory controls (79).

With regard to patients' functioning, while no statistical significance was established with the degree of obsessive–compulsive symptoms (68), average oxy-Hb concentrations were significantly positively interrelated with the global assessment of functioning (GAF) scores (57, 63) and SASS scores (47, 64, 66). For SASS in particular, observational changes in bilateral ventrolateral prefrontal cortex activity and the anterior portion of the temporal cortex (VLPFC/aTC) were associated with gains in social adaptability in MDD patients, although the MDD groups revealed less activation than the HCs in the VLPFC/aTC. Additionally, when Tsujii et al. assessed quality of life (QOL) using the Medical Outcomes Study 36-item short-form health survey (SF-36), hemodynamic responses in the prefrontal region were positively associated with the role emotional domain scores for the MDD-MF patients. Comparatively, MDD-NMF patients showed no noteworthy correlations (67). This indicated that while lower functioning was associated with lower oxy-Hb concentrations, patients with MDD-MF had qualitatively distinctive prefrontal impairment features associated with emotional role functioning unlike patients with MDD-NMF.

Pu et al. assessed coping styles with the coping inventory for stressful situations (CISS) and discovered that MDD patients were more possibly using an emotion-oriented coping style as compared to using task-orientated and avoidance-orientated coping. Emotion-oriented coping style was also positively associated with subjective assessments of the degree of depression (74). Task-orientated coping was positive correlated with regional hemodynamic changes in various prefrontal regions. The findings suggested that the hemodynamic responses in the various prefrontal regions when performing VFT imply patients who suffer from MDD utilize task-orientated coping when depressed.

Apart from the use of oxy-Hb concentration measures, three papers also assessed FC (52, 54, 72). First, in patients with affective disorders, Zhu et al. (54) showed altered patterns of FC, representing far greater disorganized patterns of correlation maps, by outlining and examining four FC types: the intraregional connectivity (SC-I), the intrahemispheric connectivity (SC-II), the symmetrically interhemispheric connectivity (LC-I), and the asymmetrical interhemispheric connectivity (LC-II). This showed that patients suffering from affective disorders indicated considerably weaker intraregional connectivity and symmetrical interhemispheric connectivity in the IFG as compared to HCs and that such disruptions in the right IFG are correlated with problems in emotional reactions and reducing negative thinking in affective disorder patients. Second, similar to previous results found by many others, Rosenbaum et al. (72) also found that the subjects who were depressed revealed increased FC in a left frontoparietal cortical network at the resting state and decreased FC in a left frontopolar cortical network while doing a performance task. However, when these relations were calculated on behalf of the separate diagnostic groups, these correlations were congruent only in the non-depressed group and varied in the depressed group, thus implying that these results might be more related to depression status than to the degree of symptoms per se. In comparison to controls, the depressed individuals indicated an exceedingly connected network with a left hemispheric focus during the resting-state condition, and this result was inferred to be related to rumination. This relationship was further examined by Rosenbaum et al. (52). Though ruminative thoughts were negatively associated with FC in the cortical areas of the default mode network (DMN), mind-wandering displayed positive correlations with FC.

Moreover, Okada et al. illustrated hemodynamic changes using brain response patterns (55). The response patterns were cerebral responses to the MDT. During the MDT, the overall response areas were analyzed by using a planimeter as the areas demarcated by the total Hb response curves and the baseline. The area deviation ratio was calculated by dividing the difference between the blood volume response areas of the right and left hemispheres by the hemisphere blood volume response area showing a lower response. When the ratios were higher (range 4–44), response patterns were classified as dominant hemispheric response patterns in normal subjects or non-dominant hemispheric response patterns in MDD patients. They demonstrated that about 50% of patients (12 out of 24 males and 4 out of 12 females) showed a “non-dominant hemisphere response pattern,” which was not seen in healthy subjects, and this “non-dominant” hemisphere could become dominant during depression. Furthermore, significant negative associations were found between the oxy-Hb and the age of onset in six channels but there were no noteworthy associations between age and oxy-Hb at the period of assessment.

### Use of fNIRS to Monitor Treatment Response

All 16 studies included in **Table 4** were case–control studies. Eleven studies were performed in Japan, one study in China, one study in Uzbekistan, one study in United Kingdom, one study in the United States, and one study in Germany. The combined sample throughout the 16 studies comprised of 282 MDD patients. Every patient met the MDD criteria of DSM-IV, DSM-5 or ICD-10. The majority of the studies examined the correlation of cerebral hemodynamic changes with improvements in the depressive symptoms of patients, while five studies (47, 48, 50, 77, 81) looked at either social and/or cognitive functioning of the patients as the treatment outcome.

**Table 4:** Summary of fNIRS studies assessing antidepressant/treatment response.

Source.	Country	Sample size (male/female)	Age (mean ± standard deviation)	Diagnostic criteria instrument	Psychopathology measure for treatment response	Treatment outcome	Medication (mg)/treatment	NIRS device/no. of channels	Duration/no. of follow-up	Paradigm	Brain area	Main finding
Tomioka et al.^2,3^ (46)	Japan	MDD: 25 (3/22)	51.9 ± 16.6	DSM-IV	HAMD	Depressive symptoms	Imipramine (118.7 ± 67.3)	52-Channel ETG-4000 (Hitachi)	12 weeks	VFT	PF, T	– NIRS signals before initiation of treatment could foretell patients' clinical response upon being treated
Onishi et al.^3^ (77).	Japan	MDD: 10 (5/5)	71.0 ± 6.0	DSM-IV	HAMD MMSE	Depressive symptoms Cognitive functioning	Mianserin (2), Sodium valproate (2), Paroxetine (2), Lithium (2), Amoxapine (1), Olanzapine (3), Milnacipran (5), Maprotiline (1)	48-Channel ETG-4000 (Hitachi)	2 follow-ups (1 day following improvement in depressive symptoms after treatment, and another day >4 weeks later)	Rock, paper, scissors (RPS)	PF	– The more left PF cortical activity tended to ↑, symptoms of depression ↓
Ohtani et al.^2,3^ (47)	Japan	MDD: 10 (4/6)	39.2 ± 12.1	DSM-IV	HAMD SASS	Social functioning	Chlorpromazine (1), Imipramine (3), Diazepam (4)	52-Channel ETG-4000 (Hitachi)	6 months	VFT	PF, T	– Longitudinal variations in SASS results were correlated positively with degree of change in the right ventrolateral PF cortex and the anterior part of the T cortex activation in MDD.
Yamagata et al. (82)	Japan	MDD: 11 (5/6)	36.3 ± 11.2	DSM-IV	HAMD	Depressive symptoms	Sertraline; week 4: (29.5 ± 10.1), week 8: (61.4 ± 20.4), week 12: (65.9 ± 23.1)	52-Channel ETG-4000 (Hitachi)	12 weeks, 3 follow-ups (weeks 4, 8, 12)	VFT	PF, T	– NIRS may be a biological marker in MDD patients for predicting clinical response to Sertraline.
Masuda et al.^2,3^ (48)	Japan	MDD: 47 Response group to SSRIs: 28 (15/13) Nonresponse group: 19 (6/13)	Response (48.9 ± 2.9) Nonresponse (43.2 ± 3.3)	DSM-IV	POMS STAI DACS	Overall functioning for response Group	Escitalopram (33), Paroxetine (7), Sertraline (5), Fluvoxamine (2)	47-Channel ETG-7100 (Hitachi)	12 weeks, weekly or biweekly follow-up	VFT	PF, T	Response to SSRI in MDD is predicted by different hemodynamic activities in the frontotempral cortex.
Feng et al.^2^ (49)	China	MDD: 15 (7/8)	30.9 ± 13.5	DSM-V	HAMD	Depressive symptoms	Music therapy, either “creative” (composing music) or “receptive” (listening to music)	45-Channel FOIRE-3000 (Shimadzu)	10 days, one session (60 min) a day	VFT,	PF	Music therapy could activate frontal cortex areas to improve mood and cognitive abilities
Aoki et al. (83)	Japan	MDD: 2 (1/1)	24.0 ± 2.0	ICD-10	NA	NA	Animal-assisted therapy (AAT) with medication	42-Channel FOIRE-3000 (Shimadzu)	Pretest and posttest design	VFT	PF	AAT helps to stimulate prefrontal activity in MDD and the effects of AAT can be evaluated by NIRS
Hirano et al.^2,3^ (50)	Japan	MDD = 30 (11/19)	59.4 ± 14.2	ICD-10	MADRS QIDS-SR MMSE	Reduction in MADRS and QIDS-SR scores (improved functioning)	Electroconvulsive therapy (ECT)	52-Channel ETG-4000 (Hitachi)	3x per week, till stable response	VFT	PF, T	Acute therapeutic effects of ECT on MDD patients is correlated to recovery from abnormal functional responses to cognitive tasks in frontal brain regions.
Takamiya et al. (84)	Japan	MDD: 33 (17/16)	46.4 ± 11.7	DSM-IV	HAMD	Differences between low-dose/high-dose groups in HAMD scores	High-dose group (> 1 defined daily dose, N =10), low-dose group (< 1 defined daily dose, N = 23)	52-Channel ETG-4000 (Hitachi)	Cross-sectional study	VFT	PF, T	The dose-dependent influence of antidepressants on NIRS signals should be considered while deciphering NIRS data.
Shinba et al. (85)	Japan	MDD: 15 (11/4)	45.4 ± 10.8	DSM-IV	MADRS	Improved functioning	Transcranial magnetic stimulation (TMS), Fluvoxamine (89.6 ± 85.8)	NIRO-3000 (Hamamatsu)	6 weeks, 5 sessions a week	NA	PF	Maintenance of frontal activation [measured by frontal hemoglobin concentration (fHbC)] during TMS stimulation is related to effectiveness of treating MDD patients.
Usami et al. (86)	Japan	MDD: 10 (1/9)	12.9 ± 0.9	DSM-IV	DSRS	Depressive symptoms Global functioning	NA	2-Channel Spectratech	6 weeks	VFT	PF	Concentration of oxy-Hb could be utilized as a state marker for changes in depressed children.
Payzieva & Maxmudova (87)	Uzbekistan	MDD: 5	NA	NA	NA	NA	NA	OxyPrem (BORL, Switzerland)	Pretest and posttest design	Mental arithmetic task	PF	Computerized cognitive exercises may help in improve cognition of MDD patients and NIRS can be used to monitor cognitive functions.
Pu et al. (81)	Japan	MDD: 29 (7/22)	72.4 ± 5.7	DSM-IV	HAMD SASS	Depression symptoms Social functioning	Paroxetine (10–40 mg, N = 15), Milnacipran (50–150 mg, N = 14)	52-Channel ETG-4000 (Hitachi)	8 weeks	VFT	PF, T	Social functioning improvements were superior in late-onset depression with initial ↓NIRS activation in the right ventrolateral PF area.
Downey et al.^2^ (51)	UK	MDD: 18	NA	NA	NA	NA	Ketamine	4 detectors and 24 sources MiniNTS (UCL)	NA	VFT	PF	PF cortical responses appear to be ↓in the severely depressed and additionally suppressed by ECT treatment
Schiffer et al. (88)	US	MDD: 10 (5/5)	35.1 ± 7.1	DSM-IV	HAMD HAMA	Depression and anxiety symptoms	4-min near-infrared (NIR) light photobiomodulation (PBM) treatment to left/right forehead	INVOS system (Somanetics)	4 weeks, 2 follow-ups (weeks 2, 4)	Lateral visual field stimulation	PF	NIR-PBM may have uses for depression treatment
Eschweiler et al. (89)	Germany	MDD: 12 (4/8)	57.0 ± 8.0	DSM-IV	HAMD BDI	Depressive symptoms	Repetitive transcranial magnetic stimulation (rTMS)	Four-site NIRS	4 weeks, 4 follow-ups (weeks 1, 2, 3, 4)	Arithmetic and mirror-tracing tasks	PF	Low local hemodynamic responses predict clinical benefits of rTMS.

^2^This article can also be found in the summary in **Table 2**.

^3^This article can also be found in the summary in **Table 3**.

MDD: major depressive disorder; HC, healthy control; HAMD, Hamilton depression rating scale; VFT, verbal fluency task; PF, prefrontal; T, temporal, F, frontal; NA, not available; MINI, Mini-international neuropsychiatric interview; DSM, Diagnostic and Statistical Manual; ICD, International Classification of Diseases; MMSE, Mini-Mental State Examination; SASS, school and staffing survey; POMS, profile of mood states; STAI, state-trait anxiety inventory; MADRS, Montgomery–Asberg depression rating scale; QIDS-SR, quick inventory of depressive symptomatology-self report; DSRS, dementia severity rating scale; HAMA, Hamilton anxiety rating scale.

Almost all patients responded favorably to treatment, as the articles reported significant improvement in social functioning or a decrease in depressive symptoms. However, the patients in the study by Takamiya et al. showed no meaningful differences in depressive scores following treatment in both the high- and low-dose groups (84). In group comparisons, there were significant outcomes on NIRS signals for high doses of antidepressants compared with low doses. This highlighted that the dose-dependent influence of antidepressants on the NIRS signal ought to be considered while understanding NIRS data. The most commonly used paradigm to stimulate cognitive activity was the VFT. One study (77) used a rock, paper, scissors (RPS) task. Two studies (87, 89) used mental arithmetic tasks, and another study (88) utilized a lateral visual field simulation. The prefrontal brain regions were examined in all of these studies.

Measuring NIRS signals before starting treatment has been shown to predict treatment progress in medication-naive patients suffering from MDD (46). In the paper by Tomioka et al. (46), the average oxy-Hb concentration values in the prefrontal and temporal regions during the VFT were significantly correlated with improvements in HAMD scores after treatment with antidepressants. The group comparisons revealed that the mean oxy-Hb concentration variations in the temporal and prefrontal cortices while performing VFT were considerably reduced in the depression group compared to HC group. This result concurred among other studies (18) that explored the uses of NIRS for the treatment of MDD.

Similar to the study by Tomioka et al. (46), Yamagata et al. also conducted a study on the application of NIRS in medication-naive participants with MDD (82). The latter study highlighted the noninvasive nature of NIRS as they conducted repeated measurements at short intervals (three follow-ups at 4-week intervals). According to their findings, they found a substantial adverse association between mean oxy-Hb concentration values in the significant cluster (at week 4) and differences in HAMD scores from weeks 4 to 8 (r = −.73) and from weeks 8 to 12 (r = −.63). As such, they also concluded that NIRS could be used in predicting MDD patients' response to antidepressant treatment.

Ohtani et al. looked at social adaptation in their study, and in their 6-month follow-up, they found that NIRS signals could be a predictor for the longitudinal assessment of social adaptation (47). Longitudinal increases in temporal and prefrontal regions were shown to be correlated with improvements in social adaptation for patients, although there was no significant change in the severity of their clinical symptoms.

On the other hand, Pu et al. investigated patients' social functioning in their study (81). The pretreatment NIRS activation in the prefrontal region was found to be positively associated with pretreatment social adaptation self-evaluation scale (SASS) scores and negatively correlated with increase in SASS scores after 8 weeks. Their discoveries suggested that NIRS signals would be useful in evaluating social functioning in patients afflicted by late-onset depression (prior to treatment) and predicting the improvement in their social functioning after treatment. Furthermore, fNIRS can be used to predict the treatment response of not only antidepressants but also other novel treatments, such as neurostimulation (50, 85, 89), music therapy (49), and animal-assisted therapy (83).

### Quality Assessment of the Included Studies

The NOS was used to assess the risk of bias in the included studies, and the results are shown in **Table 5**. No study had a complete score of eight stars. More than two-thirds of the studies had acceptable quality, of which 49 studies were awarded four or more stars, with 11 studies given seven stars, 14 studies given six stars, 19 studies given five stars, and 5 studies given four stars. Fifteen studies fared more poorly in quality with less than four stars, of which 3 studies were given three stars, 11 studies were given two stars, and 1 study was given one star.

**Table 5:** Risks of bias within studies.

Selection					Comparability	Exposure		
Study sources	Is the case definition adequate?	Representative of cases	Selection of controls	Definition of controls		Determination of exposure	Same method for determining cases and controls	Nonresponse rates
Suto et al. (18)	★			★	★	★	★	
Usami et al. (86)	★					★		
Takei et al. (63)	★		★	★	★★	★	★	
Shimodera et al. (37)	★		★	★	★	★		
Ma et al. (38)	★		★	★	★★	★	★	
Takizawa et al. (39)	★		★	★	★★	★	★	
Zhu et al. (54)	★		★	★	★★	★	★	
Matsubara et al. (42)	★		★	★	★★	★	★	
Pu et al. (81)	★				★	★	★	
Akashi et al. (43)	★			★	★	★	★	
Downey et al. (51)	★				★	★	★	
Gao et al. (44)	★		★	★	★	★	★	
Pu et al. (64)	★		★	★	★★	★	★	
Tsujii et al. (65)	★		★	★	★★	★	★	
Pu et al. (74)	★		★	★	★	★	★	
Schecklmann et al. (45)	★			★	★	★	★	
Tsujii et al. (67)	★			★	★	★	★	
Liu et al. (68)	★		★	★	★★	★	★	
Wang et al. (69)	★			★	★	★	★	
Tsujii et al. (70)	★		★	★	★	★	★	
Satomura et al. (80)	★				★	★		★
Nishida et al. (71)	★			★	★	★	★	
Rosenbaum et al. (72)	★		★	★	★	★	★	
Pu et al. (73)	★		★	★	★	★	★	
Koseiki et al. (78)	★					★		
Pu et al. (74)	★		★	★	★	★	★	
Schiffer et al. (88)	★				★	★		
Eschweiler et al. (89)	★		★	★	★	★	★	
Tomioka et al. (46)	★		★	★	★			
Pu et al. (25)	★	★		★	★★	★	★	
Matsuo et al. (26)	★			★	★★	★	★	★
Onishi et al. (77)	★					★		
Rosenbaum et al. (52)	★			★	★	★	★	★
Ohtani et al. (47)	★			★	★★	★	★	★
Herrmann et al. (27)	★	★		★	★	★	★	
Yamagata et al. (82)	★					★		
Kondo et al. (53)	★			★	★	★	★	★
Shoji et al. (28)	★			★	★	★	★	
Kinoshita et al. (29)	★					★		
Kito et al. (30)	★			★	★	★	★	
Kawano et al. (76)	★	★				★		
Matsuo et al. (31)	★	★				★		
Zhu et al. (54)	★		★	★	★	★	★	
Okada et al. (55)	★			★	★	★	★	
Uemura et al. (56)	★					★		
Matsubara et al. (32)	★			★	★	★	★	
Masuda et al. (48)	★					★		
Kinou et al. (57)	★			★	★	★	★	
Yamagata et al. (58)	★			★	★	★	★	
Koike et al. (33)	★				★			
Feng et al. (49)	★		★	★	★	★	★	
Aoki et al. (83)	★					★		
Noda et al. (59)	★			★	★	★	★	
Hirano et al. (50)	★			★	★	★	★	
Azechi et al. (34)	★			★	★	★	★	
Nishizawa et al. (60)	★			★	★	★	★	
Takamiya et al. (84)	★					★		
Matsuo et al. (35)	★			★	★★	★	★	
Ohta et al. (36)	★			★	★	★	★	
Akiyama et al. (61)	★		★	★	★	★	★	
Ohi et al. (62)	★			★	★	★	★	
Shinba et al. (85)	★					★		
Akashi et al. (79)	★							
Liske et al. (40)	★		★	★		★		★

## Discussion

Currently, there is no specific test or biomarker for diagnosing and monitoring the progression of depression. MRI studies have shown reduced size in the medial and lateral prefrontal cortex, amygdala, hippocampus, and striatum; increased functional activity in the medial prefrontal cortex, amygdala, and hippocampus; and decreased activity in the lateral prefrontal cortex and striatum in depressed subjects compared with HCs (90). Nevertheless, the applicability and utility of MRI in clinical practice is limited due to the high cost, need to stay immobile, presence of noise while scanning, long scanning duration, and risk of inducing claustrophobia. These considerations may be especially relevant for depressed patients who may be emotionally sensitive and thus find it harder to tolerate being confined to a tight space with recurrent noise for a prolonged period. Compared to fMRI, fNIRS is less expensive, fast to operate, quiet, and more portable and, thus, able to perform the scan in the clinic or ward directly and allows body movement in a naturalistic environment (91). However, some of its inherent limitations may influence the accuracy and eventual applicability in clinical practice. For instance, its spatial resolution and penetration depth are limited, which reduces access to the subcortical regions that have a pertinent role in psychiatric disorders. As fNIRS is established on the principles of neurovascular coupling, it is affected by blood pressure, hemoglobin level, blood circulation, vasculature, and carbon dioxide concentration (92, 93). Nevertheless, a meticulous study design, enhanced fNIRS techniques, and statistical processing could potentially mitigate these shortfalls.

This is the first systematic review on the application of fNIRS to depression that intended to provide an overview of the up-to-date information of 3 pertinent clinical questions: (i) the usefulness of fNIRS in differentiating depressed from healthy individuals, (ii) the correlation of fNIRS signals with depression symptomatology, and in turn (iii) monitoring treatment response. Answers to these questions are essential in ascertaining the applicability and utility of fNIRS as a tool to assist the diagnosing of depression, predicting clinical symptoms of patients, monitoring the treatment response and disease progression, and for prognosticating the disease. Based on the combined findings of the reviewed papers, most of the studies were conducted in Japan, although there has been an increasing trend for studies being conducted in China, Germany and other countries. The number of fNIRS studies has also exponentially increased year on year, suggesting an increased recognition of this technology in mainstream science. The most commonly used active paradigm is the VFT. This is not surprising as the VFT is quite a common bedside neuropsychological test that has been extensively utilized to ascertain executive function and language content (94) and is easy to perform within a short period, though it is imperative to realize that the performance on the VFT can be influenced by culture. Participants are obliged to state as many unique words that begin with a particular letter or are from a semantic category in a restricted time (up to a minute usually). Cognitive dysfunction occurs in patients who are depressed, of which impairments in executive ability is one of the core domains, in addition to learning/memory, attention/concentration, and processing speed (95). As a validated test to ascertain executive function, the VFT is able to elicit distinct differences in performance and neuroimaging responses between depressed and healthy people (23).

Based on the findings of this systematic review, the fNIRS signals show promise as an ideal biomarker in addressing our review's three fundamental questions: do fNIRS signals differentiate depressed from nondepressed individuals, correlate with specific depressive symptomatology, and in turn aid the monitoring of treatment responses (regardless of whether the treatment is a medication or another treatment modality)? The findings of our systematic review demonstrated a consistent pattern of attenuated pre-frontal stimulation in patients with depression while conducting cognitive tasks using the NIRS method, and cerebro-hemodynamic changes are associated with particular symptoms encountered by patients and treatment responses. However, most studies did not include sensitivity and specificity data that compared depressed patients from HCs, which would be important to elucidate the validity of fNIRS as a diagnostic tool. Thus, future studies may need to consider standardizing their analysis such that comparisons across studies can be made. The correlation of brain signals with clinical symptoms such as suicidal ideation, sleep quality, and psychomotor retardation may facilitate more in-depth personalized profiling of the individual's depression and may be able to better identify risk issues in management (e.g., self-harm, suicide, neglect). Symptom identification can be especially helpful when patients are not forthcoming about their symptoms, which makes it difficult to ascertain their risks. However, the majority of the selected studies had small sample populations, thereby reducing the power of the study, and there were variations in study methodologies, including the device and paradigm used, which limited the ability to effectively combine or compare results. One important aspect of fNIRS as a biomarker would be its potential to prognosticate the disease, i.e., whether the patient will improve or worsen with time. Nevertheless, the existing studies were either cross-sectional or had short longitudinal follow-ups, which made it difficult to address this issue. Longer longitudinal studies of at least 6 months to a year would be beneficial, considering that most depressive episodes last for at least a few months. Furthermore, longitudinal studies can help to elucidate whether the hemodynamic responses recorded by NIRS are a state- or trait-dependent marker of depression, as the current evidence is conflicting and confounded by several reasons.

Most studies scanned their subjects 2 weeks to 1 month after starting antidepressants, and then scans were performed monthly. Having more frequent fNIRS measurements in longitudinal studies (e.g., weekly) can provide us with a better understanding of brain dynamics and minimize the influence of confounding factors. The majority of studies to date have examined only the temporal and frontal regions. It may thus be worthwhile to expand the assessment to other brain regions, such as the occipital and parietal regions, especially because these two regions have also been implicated in depression (96, 97). Furthermore, most studies examined hemodynamic changes in discrete brain regions, with only a few studies investigating FC aberrations and even fewer ascertaining effective connectivity across brain regions (97). Connectivity studies allow a more holistic approach to how various brain areas interactively function and may represent another means of examining the disease state. Most of the studies involved patients on medication, and those with medication-naive patients had small sample sizes. Thus, we are unable to exclude the possibility that antidepressants interfered with the signal results. Studies have highlighted that antidepressants can affect the NIRS signals (84). Therefore, it may be worthwhile to have more studies involving scans of medication-naive patients, though this can be practically challenging at the ground level.

An encouraging application is combining different neuroimaging modalities to allow a more accurate and comprehensive surveillance of the neurophysiological changes. This can include combining fNIRS with EEG, fMRI, PET, or diffusion tensor imaging (DTI) to complement fNIRS. This can in turn improve the spatial and temporal resolution and thereby increase sensitivity and specificity. Nevertheless, the applicability of combining modalities such as fMRI and PET is limited due to the immobile and bulky apparatus, and although EEG is portable, use of EEG increases the setup time, and the increased noise-signal ratio with artifacts may reduce its accuracy. On the other hand, integrative approaches that combine various modalities of biomarkers such as genetic, neuroimaging, and neuropsychological data that are analyzed using high-dimensional multivariate statistical methods are gaining prominence and may more comprehensively facilitate disease diagnostics and prediction (98).

Our review has a number of limitations. First, we selected observational studies that had small sample sizes, thereby increasing the risk of selective and confounding biases and reduced power due to small effect sizes. However, based on our search, there were no randomized controlled trials of patients with depression using fNIRS. Second, we only included studies in English and searched in four databases. We recognize that there are an increasing number of fNIRS studies conducted in China and other countries that may not be published in English and indexed in these four databases. Thus, this potentially led to publication bias. Third, we also included conference proceedings, and several details of these datasets were unavailable from the source. However, it was essential to include them to reflect the spectrum of studies being performed in the current fNIRS research landscape. Regardless, this review has strengths in being the first to combine three pertinent questions pertaining to fNIRS as a biomarker, and there has not been an updated systematic review of fNIRS in depression since 2015. This study is a timely update of the current landscape of using fNIRS on depressed patients, given the increasing availability of this technology worldwide.

In conclusion, there is a good amount of evidence in the current literature to suggest fNIRS as a diagnostic and predictive tool for MDD, and it has shown consistent hemodynamic patterns in depressed patients compared to healthy individuals. There is also an increasing amount of studies on fNIRS in depression, indicating the increased recognition of this technique in the study of depression. Future studies involving larger sample sizes, standardized methodology, examination of more brain regions in an integrative approach, and longitudinal follow-ups are needed to advance the use of fNIRS in psychiatric clinical practice and research.

## Author Contributions

Conceptualization: CH. Data extraction and review: LL, AL, NC, RT, SL. Writing: CH. Review and manuscript amendment: LL, AL, NC, RT, SL, RH.

## Conflict of Interest

The authors declare that the research was conducted in the absence of any commercial or financial relationships that could be construed as a potential conflict of interest.

## References

[1] World Health Organization (2017). Depression and Other Common Mental Disorders: Global Health Estimates. World Health Organization [cited 2019 Oct 13]; Available from: https://apps.who.int/iris/bitstream/handle/10665/254610/WHO-MSD-MER-2017.2-eng.pdf?sequence=1.

[2] Lim GY Tam WW Lu Y Ho CS Zhang MW Ho RC Prevalence of Depression in the Community from 30 Countries between 1994 and 2014. Sci Rep (2018) 12 8(1):2861. 10.1038/s41598-018-21243-x PMC5809481 29434331

[3] Choo CC Harris KM Ho RC Prediction of Lethality in Suicide Attempts: Gender Matters. Omega. (2019) 80(1):87–103. 10.1177/0030222817725182 28828921

[4] Sheehan DV Depression: underdiagnosed, undertreated, underappreciated. Manag Care Langhorne Pa (2004) 13(6 Suppl Depression):6–8. 15293765

[5] Mayberg HS Modulating dysfunctional limbic-cortical circuits in depression: towards development of brain-based algorithms for diagnosis and optimised treatment. Br Med Bull (2003) 65:193–207. 10.1093/bmb/65.1.193 12697626

[6] Ottowitz WE Dougherty DD Savage CR The neural network basis for abnormalities of attention and executive function in major depressive disorder: implications for application of the medical disease model to psychiatric disorders. Harv Rev Psychiatry (2002) 10(2):86–99. 10.1080/10673220216210 11897749

[7] Fava M Diagnosis and definition of treatment-resistant depression. Biol Psychiatry (2003) 53(8):649–59. 10.1016/S0006-3223(03)00231-2 12706951

[8] Gaynes BN Warden D Trivedi MH Wisniewski SR Fava M Rush AJ What did STAR*D teach us? Results from a large-scale, practical, clinical trial for patients with depression. Psychiatr Serv Wash DC. (2009) 60(11):1439–45. 10.1176/ps.2009.60.11.1439 19880458

[9] Olchanski N McInnis Myers M Halseth M Cyr PL Bockstedt L Goss TF The economic burden of treatment-resistant depression. Clin Ther (2013) 35(4):512–22. 10.1016/j.clinthera.2012.09.001 23490291

[10] Strawbridge R Young AH Cleare AJ Biomarkers for depression: recent insights, current challenges and future prospects. Neuropsychiatr Dis Treat (2017) 13:1245–62. 10.2147/NDT.S114542 PMC5436791 28546750

[11] Okada G Okamoto Y Yamashita H Ueda K Takami H Yamawaki S Attenuated prefrontal activation during a verbal fluency task in remitted major depression. Psychiatry Clin Neurosci (2009) 63(3):423–5. 10.1111/j.1440-1819.2009.01952.x 19566776

[12] Meyer JH Houle S Sagrati S Carella A Hussey DF Ginovart N Brain serotonin transporter binding potential measured with carbon 11-labeled DASB positron emission tomography: effects of major depressive episodes and severity of dysfunctional attitudes. Arch Gen Psychiatry (2004) 61(12):1271–9. 10.1001/archpsyc.61.12.1271 15583118

[13] Drevets WC Neuroimaging studies of mood disorders. Biol Psychiatry (2000) 48(8):813–29. 10.1016/S0006-3223(00)01020-9 11063977

[14] Lai CYY Ho CSH Lim CR Ho RCM Functional near-infrared spectroscopy in psychiatry. BJPsych Adv (2017) 23(5):324–30. 10.1192/apt.bp.115.015610

[15] Fukuda M Near-infrared spectroscopy in psychiatry. Brain Nerve Shinkei Kenkyu No Shinpo. (2012) 64(2):175–83. 22308262

[16] Scholkmann F Kleiser S Metz AJ Zimmermann R Mata Pavia J Wolf U A review on continuous wave functional near-infrared spectroscopy and imaging instrumentation and methodology. NeuroImage (2014) 85 (Pt 1):6–27. 10.1016/j.neuroimage.2013.05.004 23684868

[17] Boas DA Elwell CE Ferrari M Taga G Twenty years of functional near-infrared spectroscopy: introduction for the special issue. NeuroImage (2014) 85 Pt 1:1–5. 10.1016/j.neuroimage.2013.11.033 24321364

[18] Suto T Fukuda M Ito M Uehara T Mikuni M Multichannel near-infrared spectroscopy in depression and schizophrenia: cognitive brain activation study. Biol Psychiatry (2004) 55(5):501–11. 10.1016/j.biopsych.2003.09.008 15023578

[19] Noda T Nakagome K Setoyama S Matsushima E Working memory and prefrontal/temporal hemodynamic responses during post-task period in patients with schizophrenia: A multi-channel near-infrared spectroscopy study. J Psychiatr Res (2017) 95:288–98. 10.1016/j.jpsychires.2017.09.001 28934615

[20] Hirose T Tsujii N Mikawa W Shirakawa O Delayed hemodynamic responses associated with a history of suicide attempts in bipolar disorder: a multichannel near-infrared spectroscopy study. Psychiatry Res Neuroimaging. (2018) 280:15–21. 10.1016/j.pscychresns.2018.08.003 30125755

[21] Katzorke A Zeller JBM Müller LD Lauer M Polak T Deckert J Decreased hemodynamic response in inferior frontotemporal regions in elderly with mild cognitive impairment. Psychiatry Res Neuroimaging. (2018) 274:11–8. 10.1016/j.pscychresns.2018.02.003 29472145

[22] Ueda S Ota T Iida J Yamamuro K Yoshino H Kishimoto N Reduced prefrontal hemodynamic response in adult attention-deficit hyperactivity disorder as measured by near-infrared spectroscopy. Psychiatry Clin Neurosci (2018) 72(6):380–90. 10.1111/pcn.12643 29405508

[23] Zhang H Dong W Dang W Quan W Tian J Chen R Near-infrared spectroscopy for examination of prefrontal activation during cognitive tasks in patients with major depressive disorder: a meta-analysis of observational studies. Psychiatry Clin Neurosci (2015) 69(1):22–33. 10.1111/pcn.12209 24897940

[24] Wells GA Shea B O'Connell D Peterson J Welch V Losos M (2000). The Newcastle– Ottawa Scale (NOS) for assessing the quality of nonrandomized studies in meta-analyses. [Internet]. [cited 2019 Oct 13]. Available from: http://www.ohri.ca/programs/clinical_epidemiology/oxford.asp.

[25] Pu S Yamada T Yokoyama K Matsumura H Kobayashi H Sasaki N A multi-channel near-infrared spectroscopy study of prefrontal cortex activation during working memory task in major depressive disorder. Neurosci Res (2011) 70(1):91–7. 10.1016/j.neures.2011.01.001 21241745

[26] Matsuo K Kato T Fukuda M Kato N Alteration of Hemoglobin Oxygenation in the Frontal Region in Elderly Depressed Patients as Measured by Near-infrared Spectroscopy. J Neuropsychiatry Clin Neurosci (2000) 12(4):465–71. 10.1176/jnp.12.4.465 11083163

[27] Herrmann MJ Ehlis A-C Fallgatter AJ Bilaterally Reduced Frontal Activation During a Verbal Fluency Task in Depressed Patients as Measured by Near-Infrared Spectroscopy. J Neuropsychiatry Clin Neurosci (2004) 16(2):170–5. 10.1176/jnp.16.2.170 15260368

[28] Shoji Y Morita K Yanagimoto H Fujiki R Ishii Y Uchimura N Characteristics of the single event related [Oxy-Hb] changes in patients with depressive disorder.

[29] Kinoshita S Kanazawa T Kikuyama H Yoneda H Clinical application of DEX/CRH test and multi-channel NIRS in patients with depression. Behav Brain Funct BBF. (2016) 12(1):25. 10.1186/s12993-016-0108-x 27582123 PMC5007847

[30] Kito H Ryokawa A Kinoshita Y Sasayama D Sugiyama N Ogihara T Comparison of alterations in cerebral hemoglobin oxygenation in late life depression and Alzheimer's disease as assessed by near-infrared spectroscopy. Behav Brain Funct BBF (2014) 10:8. 10.1186/1744-9081-10-8 24636630 PMC3995325

[31] Matsuo K Kato N Kato T Decreased cerebral haemodynamic response to cognitive and physiological tasks in mood disorders as shown by near-infrared spectroscopy. Psychol Med (2002) 32(6):1029–37. 10.1017/S0033291702005974 12214784

[32] Matsubara T Matsuo K Harada K Nakashima M Nakano M Hirotsu M Different Fronto-Temporal Activation During an Emotional Word Task in Patients with Unipolar and Bipolar Depression: A Functional Near-Infrared Spectroscopy Study (2015). Biol Psychiatry (2015) 77:1S–444S.

[33] Koike S Sakakibara E Satomura Y Sakurada H Yamagishi M Matsuoka J Differentiation between Schizophrenia, Bipolar Disorder, and Major Depression Using the Prefrontal Brain Activity and the Evaluation of Ultra-high Risk for Psychosis: A Large-Sample Functional Near-infrared Spectroscopy Study. Early Interv Psychiatry (2018) 12(Suppl. 1):104–232. 10.1111/eip.12724

[34] Azechi M Iwase M Ishii R Ikezawa K Canuet L Kurimoto R P27-5 Frontal lobe dysfunction and regional hemodynamic changes in major depression: A near infrared spectroscopy study. Clin Neurophysiol (2010) 121:S264. 10.1016/S1388-2457(10)61079-6

[35] Matsuo K Onodera Y Hamamoto T Muraki K Kato N Kato T Hypofrontality and microvascular dysregulation in remitted late-onset depression assessed by functional near-infrared spectroscopy. NeuroImage. (2005) 26(1):234–42. 10.1016/j.neuroimage.2005.01.024 15862223

[36] Ohta H Yamagata B Tomioka H Takahashi T Yano M Nakagome K Hypofrontality in panic disorder and major depressive disorder assessed by multi-channel near-infrared spectroscopy. Depress Anxiety (2008) 25(12):1053–9. 10.1002/da.20463 18833572

[37] Shimodera S Imai Y Kamimura N Morokuma I Fujita H Inoue S Near-infrared spectroscopy of bipolar disorder may be distinct from that of unipolar depression and of healthy controls: NIRS of bipolar vs unipolar disorder. Asia-Pac Psychiatry (2012) 4(4):258–65. 10.1111/j.1758-5872.2012.00218.x

[38] Ma X-Y Wang Y-J Xu B Feng K Sun G-X Zhang X-Q Near-Infrared Spectroscopy Reveals Abnormal Hemodynamics in the Left Dorsolateral Prefrontal Cortex of Menopausal Depression Patients. Dis Markers (2017) 2017:1695930. 10.1155/2017/1695930 28293062 PMC5331417

[39] Takizawa R Fukuda M Kawasaki S Kasai K Mimura M Pu S Neuroimaging-aided differential diagnosis of the depressive state. NeuroImage (2014) 85 (Pt 1):498–507. 10.1016/j.neuroimage.2013.05.126 23764293

[40] Liske BCJ Ackermann PH Münch D Florian H Ehlis A-C Stevens A New electrophysiological findings in the detection of malingering attention deficits in subjects with an episode of depression compared to healthy subjects. Clin Neurophysiology (2015) 126:e63–e170. 10.1016/j.clinph.2015.04.178

[41] Zhu Y Quan W Wang H Ma Y Yan J Zhang H Prefrontal activation during a working memory task differs between patients with unipolar and bipolar depression: A preliminary exploratory study. J Affect Disord (2018) 225:64–70. 10.1016/j.jad.2017.07.031 28797920

[42] Matsubara T Matsuo K Nakashima M Nakano M Harada K Watanuki T Prefrontal activation in response to emotional words in patients with bipolar disorder and major depressive disorder. NeuroImage (2014) 85 (Pt 1):489–97. 10.1016/j.neuroimage.2013.04.098 23643923

[43] Akashi H Tsujii N Mikawa W Adachi T Kirime E Shirakawa O Prefrontal cortex activation is associated with a discrepancy between self- and observer-rated depression severities of major depressive disorder: a multichannel near-infrared spectroscopy study. J Affect Disord (2015) 174:165–72. 10.1016/j.jad.2014.11.020 25497474

[44] Gao L Cai Y Wang H Wang G Zhang Q Yan X Probing prefrontal cortex hemodynamic alterations during facial emotion recognition for major depression disorder through functional near-infrared spectroscopy. J Neural Eng. (2019) 16(2):026026. 10.1088/1741-2552/ab0093 30669122

[45] Schecklmann M Dresler T Beck S Jay JT Febres R Haeusler J Reduced prefrontal oxygenation during object and spatial visual working memory in unpolar and bipolar depression. Psychiatry Res (2011) 194(3):378–84. 10.1016/j.pscychresns.2011.01.016 22079657

[46] Tomioka H Yamagata B Kawasaki S Pu S Iwanami A Hirano J A Longitudinal Functional Neuroimaging Study in Medication-Naïve Depression after Antidepressant Treatment. PloS One (2015) 10(3):e0120828. 10.1371/journal.pone.0120828 25786240 PMC4364958

[47] Ohtani T Nishimura Y Takahashi K Ikeda-Sugita R Okada N Okazaki Y Association between longitudinal changes in prefrontal hemodynamic responses and social adaptation in patients with bipolar disorder and major depressive disorder. J Affect Disord (2015) 176:78–86. 10.1016/j.jad.2015.01.042 25702603

[48] Masuda K Nakanishi M Okamoto K Kawashima C Oshita H Inoue A Different functioning of prefrontal cortex predicts treatment response after a selective serotonin reuptake inhibitor treatment in patients with major depression. J Affect Disord (2017) 214:44–52. 10.1016/j.jad.2017.02.034 28266320

[49] Feng K Shen C-Y Ma X-Y Chen G-F Zhang M-L Xu B Effects of music therapy on major depressive disorder: A study of prefrontal hemodynamic functions using fNIRS. Psychiatry Res (2019) 275:86–93. 10.1016/j.psychres.2019.03.015 30884335

[50] Hirano J Takamiya A Yamagata B Hotta S Miyasaka Y Pu S Frontal and temporal cortical functional recovery after electroconvulsive therapy for depression: A longitudinal functional near-infrared spectroscopy study. J Psychiatr Res (2017) 91:26–35. 10.1016/j.jpsychires.2017.02.018 28292650

[51] Downey D Sabrina B Liam T, Rebecca E Elwell C Richard M-W Prefrontal cortex haemodynamic responses in severe major depression and the effects of ECT: an fNIRS study. Eur Neuropsychopharmacol (2016), S304.

[52] Rosenbaum D Haipt A Fuhr K Haeussinger FB Metzger FG Nuerk H-C Aberrant functional connectivity in depression as an index of state and trait rumination. Sci Rep (2017) 7(1):2174. 10.1038/s41598-017-02277-z 28526867 PMC5438394

[53] Kondo A Shoji Y Morita K Sato M Ishii Y Yanagimoto H Characteristics of oxygenated hemoglobin concentration change during pleasant and unpleasant image-recall tasks in patients with depression: Comparison with healthy subjects. Psychiatry Clin Neurosci (2018) 72(8):611–22. 10.1111/pcn.12684 29808572

[54] Zhu H Xu J Li J Peng H Cai T Li X Decreased functional connectivity and disrupted neural network in the prefrontal cortex of affective disorders: A resting-state fNIRS study. J Affect Disord (2017) 221:132–44. 10.1016/j.jad.2017.06.024 28645025

[55] Okada F Takahashi N Tokumitsu Y Dominance of the “nondominant” hemisphere in depression. J Affect Disord (1996) 37(1):13–21. 10.1016/0165-0327(95)00040-2 8682974

[56] Uemura K Shimada H Doi T Makizako H Park H Suzuki T Depressive symptoms in older adults are associated with decreased cerebral oxygenation of the prefrontal cortex during a trail-making test. Arch Gerontol Geriatr. (2014) 59(2):422–8. 10.1016/j.archger.2014.07.003 25064032

[57] Kinou M Takizawa R Marumo K Kawasaki S Kawakubo Y Fukuda M Differential spatiotemporal characteristics of the prefrontal hemodynamic response and their association with functional impairment in schizophrenia and major depression. Schizophr Res (2013) 150(2–3):459–67. 10.1016/j.schres.2013.08.026 24016725

[58] Yamagata B Tomioka H Takahashi T Isomura AJ Kobayashi H Mimura M Differentiating early and late-onset depression with multichannel near-infrared spectroscopy. Psychogeriatrics. (2008) 8(2):79–87. 10.1111/j.1479-8301.2008.00232.x

[59] Noda T Yoshida S Matsuda T Okamoto N Sakamoto K Koseki S Frontal and right temporal activations correlate negatively with depression severity during verbal fluency task: a multi-channel near-infrared spectroscopy study. J Psychiatr Res (2012) 46(7):905–12. 10.1016/j.jpsychires.2012.04.001 22572569

[60] Nishizawa Y Kanazawa T Kawabata Y Matsubara T Maruyama S Kawano M fNIRS Assessment during an Emotional Stroop Task among Patients with Depression: Replication and Extension. Psychiatry Investig (2019) 16(1):80–6. 10.30773/pi.2018.11.12.2 PMC6354038 30696239

[61] Akiyama T Koeda M Okubo Y Kimura M Hypofunction of left dorsolateral prefrontal cortex in depression during verbal fluency task: A multi-channel near-infrared spectroscopy study. J Affect Disord (2018) 231:83–90. 10.1016/j.jad.2018.01.010 29455100

[62] Ohi K Shimada T Kihara H Yasuyama T Sawai K Matsuda Y Impact of Familial Loading on Prefrontal Activation in Major Psychiatric Disorders: A Near-Infrared Spectroscopy (NIRS) Study. Sci Rep (2017) 7:44268. 10.1038/srep44268 28295013 PMC5353718

[63] Takei Y Suda M Aoyama Y Sakurai N Tagawa M Motegi T Near-infrared spectroscopic study of frontopolar activation during face-to-face conversation in major depressive disorder and bipolar disorder. J Psychiatr Res (2014) 57:74–83. 10.1016/j.jpsychires.2014.06.009 25056175

[64] Pu S Matsumura H Yamada T Ikezawa S Mitani H Adachi A Reduced frontopolar activation during verbal fluency task associated with poor social functioning in late-onset major depression: Multi-channel near-infrared spectroscopy study. Psychiatry Clin Neurosci (2008) 62(6):728–37. 10.1111/j.1440-1819.2008.01882.x 19068011

[65] Tsujii N Mikawa W Tsujimoto E Adachi T Niwa A Ono H Reduced left precentral regional responses in patients with major depressive disorder and history of suicide attempts. PloS One (2017) 12(4):e0175249. 10.1371/journal.pone.0175249 28380030 PMC5381916

[66] Pu S Yamada T Yokoyama K Matsumura H Mitani H Adachi A Reduced prefrontal cortex activation during the working memory task associated with poor social functioning in late-onset depression: multi-channel near-infrared spectroscopy study. Psychiatry Res (2012) 203(2–3):222–8. 10.1016/j.pscychresns.2012.01.007 22964135

[67] Tsujii N Mikawa W Tsujimoto E Akashi H Adachi T Kirime E Relationship between prefrontal hemodynamic responses and quality of life differs between melancholia and non-melancholic depression. Psychiatry Res Neuroimaging. (2016) 253:26–35. 10.1016/j.pscychresns.2016.04.015 27259838

[68] Liu X Sun G Zhang X Xu B Shen C Shi L Relationship between the prefrontal function and the severity of the emotional symptoms during a verbal fluency task in patients with major depressive disorder: a multi-channel NIRS study. Prog Neuropsychopharmacol Biol Psychiatry (2014) 54:114–21. 10.1016/j.pnpbp.2014.05.005 24842802

[69] Wang J Lv B Quan W Wydell TN Tian J Wang P Right fronto-temporal activation differs between Chinese first-episode and recurrent Major Depression Disorders during a verbal fluency task: A near-infrared spectroscopy study. Psychiatry Res Neuroimaging. (2017) 264:68–75. 10.1016/j.pscychresns.2017.03.013 28463749

[70] Tsujii N Mikawa W Akashi H Tsujimoto E Adachi T Kirime E Right temporal activation differs between melancholia and nonmelancholic depression: a multichannel near-infrared spectroscopy study. J Psychiatr Res (2014) 55:1–7. 10.1016/j.jpsychires.2014.04.003 24780385

[71] Nishida M Kikuchi S Matsumoto K Yamauchi Y Saito H Suda S Sleep complaints are associated with reduced left prefrontal activation during a verbal fluency task in patients with major depression: A multi-channel near-infrared spectroscopy study. J Affect Disord (2017) 207:102–9. 10.1016/j.jad.2016.09.028 27721182

[72] Rosenbaum D Hagen K Deppermann S Kroczek AM Haeussinger FB Heinzel S State-dependent altered connectivity in late-life depression: a functional near-infrared spectroscopy study. Neurobiol Aging. (2016) 39:57–68. 10.1016/j.neurobiolaging.2015.11.022 26923402

[73] Pu S Nakagome K Yamada T Yokoyama K Matsumura H Yamada S Suicidal ideation is associated with reduced prefrontal activation during a verbal fluency task in patients with major depressive disorder. J Affect Disord (2015) 181:9–17. 10.1016/j.jad.2015.04.010 25913539

[74] Pu S Nakagome K Yamada T Yokoyama K Matsumura H Mitani H The relationship between the prefrontal activation during a verbal fluency task and stress-coping style in major depressive disorder: a near-infrared spectroscopy study. J Psychiatr Res (2012) 46(11):1427–34. 10.1016/j.jpsychires.2012.08.001 22935269

[75] Alexopoulos GS Depression in the elderly. Lancet Lond Engl (2005) 365(9475):1961–70. 10.1016/S0140-6736(05)66665-2 15936426

[76] Kawano M Kanazawa T Kikuyama H Tsutsumi A Kinoshita S Kawabata Y Correlation between frontal lobe oxy-hemoglobin and severity of depression assessed using near-infrared spectroscopy. J Affect Disord (2016) 205:154–8. 10.1016/j.jad.2016.07.013 27449547

[77] Onishi Y Kikuchi S Watanabe E Kato S Alterations in prefrontal cortical activity in the course of treatment for late-life depression as assessed on near-infrared spectroscopy. Psychiatry Clin Neurosci (2008) 62(2):177–84. 10.1111/j.1440-1819.2008.01752.x 18412840

[78] Koseki S Noda T Yokoyama S Kunisato Y Ito D Suyama H The relationship between positive and negative automatic thought and activity in the prefrontal and temporal cortices: a multi-channel near-infrared spectroscopy (NIRS) study. J Affect Disord (2013) 151(1):352–9. 10.1016/j.jad.2013.05.067 23829998

[79] Akashi H Noa T Sakai S Mikawa W Kirime E Shirakawa O Inhibitory controls in bipolar and major depressive disorder: a NIRS study. Bipolar Disord (2012) 14:52.

[80] Satomura Y Sakakibara E Takizawa R Koike S Nishimura Y Sakurada H Severity-dependent and -independent brain regions of major depressive disorder: A long-term longitudinal near-infrared spectroscopy study. J Affect Disord (2019) 243:249–54. 10.1016/j.jad.2018.09.029 30248636

[81] Pu S Nakagome K Yamada T Yokoyama K Matsumura H Nagata I Prefrontal activation predicts social functioning improvement after initial treatment in late-onset depression. J Psychiatr Res (2015) 62:62–70. 10.1016/j.jpsychires.2015.01.009 25659188

[82] Yamagata B Yamanaka K Takei Y Hotta S Hirano J Tabuchi H Brain functional alterations observed 4-weekly in major depressive disorder following antidepressant treatment. J Affect Disord (2019) 252:25–31. 10.1016/j.jad.2019.04.001 30959413

[83] Aoki J Iwahashi K Ishigooka J Fukamauchi F Numajiri M Ohtani N Evaluation of cerebral activity in the prefrontal cortex in mood [affective] disorders during animal-assisted therapy (AAT) by near-infrared spectroscopy (NIRS): A pilot study. Int J Psychiatry Clin Pract (2012) 16(3):205–13. 10.3109/13651501.2011.644565 22486555

[84] Takamiya A Hirano J Ebuchi Y Ogino S Shimegi K Emura H High-dose antidepressants affect near-infrared spectroscopy signals: A retrospective study. NeuroImage Clin (2017) 14:648–55. 10.1016/j.nicl.2017.02.008 PMC5357702 28348956

[85] Shinba T Kariya N Matsuda S Matsuda H Obara Y Increase of frontal cerebral blood volume during transcranial magnetic stimulation in depression is related to treatment effectiveness: A pilot study with near-infrared spectroscopy. Psychiatry Clin Neurosci (2018) 72(8):602–10. 10.1111/pcn.12680 29774621

[86] Usami M Iwadare Y Kodaira M Watanabe K Saito K Near Infrared Spectroscopy Study of the Frontopolar Hemodynamic Response and Depressive Mood in Children with Major Depressive Disorder: A Pilot Study. Yoshikawa T, editor. PloS One (2014) 9(1):e86290. 10.1371/journal.pone.0086290 24466008 PMC3900510

[87] Payzieva S Maxmudova D NIRS Study of the Effects of Computerized Brain Training Games for Cognitive Rehabilitation of Major Depressive Disorder Patients in Remission: A Pilot Study. Stud Health Technol Inform. (2014) 199:163–7. 24875713

[88] Schiffer F Johnston AL Ravichandran C Polcari A Teicher MH Webb RH Psychological benefits 2 and 4 weeks after a single treatment with near infrared light to the forehead: a pilot study of 10 patients with major depression and anxiety. Behav Brain Funct BBF. (2009) 5:46. 10.1186/1744-9081-5-46 19995444 PMC2796659

[89] Eschweiler GW Wegerer C Schlotter W Spandl C Stevens A Bartels M Left prefrontal activation predicts therapeutic effects of repetitive transcranial magnetic stimulation (rTMS) in major depression. Psychiatry Res (2000) 99(3):161–72. 10.1016/S0925-4927(00)00062-7 11068197

[90] Arnone D McIntosh AM Ebmeier KP Munafò MR Anderson IM Magnetic resonance imaging studies in unipolar depression: systematic review and meta-regression analyses. Eur Neuropsychopharmacol J Eur Coll Neuropsychopharmacol (2012) 22(1):1–16. 10.1016/j.euroneuro.2011.05.003 21723712

[91] Ho CSH Zhang MWB Ho RCM Optical Topography in Psychiatry: A Chip Off the Old Block or a New Look Beyond the Mind-Brain Frontiers? Front Psychiatry (2016) 7:74. 10.3389/fpsyt.2016.00074 27199781 PMC4844608

[92] Kirilina E Jelzow A Heine A Niessing M Wabnitz H Brühl R The physiological origin of task-evoked systemic artefacts in functional near infrared spectroscopy. NeuroImage. (2012) 61(1):70–81. 10.1016/j.neuroimage.2012.02.074 22426347 PMC3348501

[93] Caldwell M Scholkmann F Wolf U Wolf M Elwell C Tachtsidis I Modelling confounding effects from extracerebral contamination and systemic factors on functional near-infrared spectroscopy. NeuroImage. (2016) 143:91–105. 10.1016/j.neuroimage.2016.08.058 27591921 PMC5139986

[94] Hanlly JR Dewick HC Davies ADM Playeer J Turnbull C Verbal fluency in parkinson's disease. Neuropsychologia. (1990) 28(7):737–41. 10.1016/0028-3932(90)90129-C 2215885

[95] Zuckerman H Pan Z Park C Brietzke E Musial N Shariq AS Recognition and Treatment of Cognitive Dysfunction in Major Depressive Disorder. Front Psychiatry (2018) 9:655. 10.3389/fpsyt.2018.00655 30564155 PMC6288549

[96] Freedman M Frontal and parietal lobe dysfunction in depression: delayed alternation and tactile learning deficits. Neuropsychologia (1994) 32(8):1015–25. 10.1016/0028-3932(94)90050-7 7969863

[97] Li J Xu C Cao X Gao Q Wang Y Wang Y Abnormal activation of the occipital lobes during emotion picture processing in major depressive disorder patients. Neural Regener Res (2013) 8(18):1693–701. 10.3969/j.issn.1673-5374.2013.18.007 PMC4145913 25206466

[98] Mas S Gassó P Morer A Calvo A Bargalló N Lafuente A Integrating Genetic, Neuropsychological and Neuroimaging Data to Model Early-Onset Obsessive Compulsive Disorder Severity. PloS One (2016) 11(4):e0153846. 10.1371/journal.pone.0153846 27093171 PMC4836736

